# How to tackle complexity in urban climate resilience? Negotiating climate science, adaptation and multi-level governance in India

**DOI:** 10.1371/journal.pone.0253904

**Published:** 2021-07-01

**Authors:** Mahendra Sethi, Richa Sharma, Subhakanta Mohapatra, Shilpi Mittal

**Affiliations:** 1 Technical University of Berlin, Berlin, Germany; 2 Mercator Research Institute on Global Commons and Climate Change, Berlin, Germany; 3 Indian Society for Applied Research & Development, New Delhi, India; 4 India Public Health Foundation of India, New Delhi, India; 5 School of Sciences, Indira Gandhi National Open University, New Delhi, India; 6 G.D. Goenka University, Gurugram, Haryana, India; Shenzhen University, CHINA

## Abstract

As the world’s population is expected to be over 2/3^rd^ urban by 2050, climate action in cities is a growing area of interest in the inter-disciplines of development policy, disaster mitigation and environmental governance. The climate impacts are expected to be quite severe in the developing world, given its urban societies are densely packed, vastly exposed to natural elements while possessing limited capabilities. There is a notable ambiguity and complexity that inhibits a methodical approach in identifying urban resilience measures. The complexity is due to intersection of large number of distinct variables in *climate geoscience* (precipitation and temperature anomalies at different locations, RCPs, timeline), *adaptation alternatives* (approach, priority, intervention level) and *urban governance* (functional mandate, institutional capacity, and plans & policies). This research examines how disparate and complex knowledge and information in these inter-disciplines can be processed for systematic ‘negotiation’ to situate, ground and operationalize resilience in cities. With India as a case, we test this by simulating mid-term and long-run climate scenarios (2050 & 2080) to map regional climate impacts that shows escalation in the intensity of climate events like heat waves, urban flooding, landslides and sea level rise. We draw on suitable adaptation measures for five key urban sectors- water, infrastructure (including energy), building, urban planning, health and conclude a sleuth of climate resilience building measures for policy application through national/ state policies, local urban plans and preparation of city resilience strategy, as well as advance the research on ‘negotiated resilience’ in urban areas

## 1. Introduction

Climate action in urban areas is a growing area of interest in the inter-disciplines of development policy, disaster mitigation and environmental governance [1–3]. By 2050, the world’s population is expected to be 68% urban [4], flaring concerns on how rapidly growing, yet financially incapacitated cities would respond to climate impacts, particularly in the developing world [5–7]. These impacts would be quite severe in Asia, given its urban societies are densely packed, vastly exposed to natural elements while possessing limited capabilities [8–10]. The last decade has witnessed a growing role of cities in the global energy, environment and sustainability policies [3, 11–14], yet the effective pursuit of urban resilience in developing contexts lacks sufficient scientific rigor and policy clarity at the implementation level. The United Nations Framework Convention on Climate Change (UNFCCC) in its last two scientific assessments, most notably in AR5 [3], exhaustively covered global policies and scholarly literature on how urban systems worldwide are combating climate impacts, the intervening barriers and knowledge gaps. Despite the original focus on mitigation in science-policy discourse in urban systems [15, 16], latest bibliometric analysis of urban climate change literature shows that adaptation is now receiving considerable attention [17]. The local climate plans usually identify adaptation pathways for specific climate hazards in an urban sector for e.g. floods. An assessment of climate action tools relevant to urban areas reports that 39% urban climate tools are adaptation centric [18].

In practice, adaptation and resilience accounts for a marginal 12.1% of the global urban initiatives [19], although the need of having a science-based policy approach in greater understanding urban vulnerabilities and shaping appropriate responses has been constantly emphasized [20–22]. There is a notable ambiguity and complexity that inhibits a methodical approach in identifying public management of urban resilience measures. The complexity is due to intersection of large number of distinct variables in *climate geoscience* (precipitation and temperature anomalies at different locations, RCPs, timeline), *adaptation alternatives* (approach, priority, intervention level) and *urban governance* (functional mandate, institutional capacity, and plans & policies). This creates an ambiguity in answering the following key research-policy questions: (a) what are the downscaled climate scenarios and how these perpetuate regional vulnerabilities? (b) how to identify and prioritize adaptation options across different sectors?, (c) how to strategically integrate climate actions with multi-level policies and governance, particularly at the urban level.?

The complex situation necessitates a planned and methodical approach in devising a climate-resilient urban future [23] that underscores the process of ‘negotiation’ to situate, ground and operationalize ‘resilience’. The concept puts particular accent on the procedural orientation of resilience–it is not something that ‘exists’ and that we can uniformly define, rather it is a process that requires engagement with diverse actors and interests, both in specific places and across scales. We internalize this process of engagement in the research methodology by examining how disparate and complex knowledge and information in the inter-disciplines of climate geoscience, adaptation, and policy & governance be systematically processed to arrive at urban resilience measures. We test this by mapping mid-term and long-run climate scenarios (2050 & 2080) in a rapidly developing context like India as a case to map regional climate impacts, evaluate plausible adaptation measures to negotiate suitable urban resilience mechanisms within the current multi-level governance system. Unlike more technocratic (top-down) approaches in evaluating climate responses, our approach engages with their diverse interests, mandates, policies and capacities of local stakeholders to deal with climate resilience issues evident across the national and urban landscape.

Climate change impacts in India are already being felt and impacting human life and livelihoods of many. Hundreds of people are losing their lives and falling ill due to the intensive heat waves during summers every year [24]. The population in Indian cities is vulnerable to floods that are detrimental to the economy, lives, and livelihoods, and thousands are being rendered homeless while losing their possessions and assets [7]. The intense rain and extensive flash flood episodes in Mumbai, Srinagar, Chennai, Bengaluru, etc. in the last one decade have revealed what mayhem and misery climate hazards could unfold in large settlements. Being prepared and resilient to climate change should therefore be a high priority for cities when planning for development and this requires a better understanding of climate impacts and actions at different levels- national, state, regional, local, neighbourhood, building and individual level. The complete absence of a nation-wide urbanization policy and the uncertainties in local implementation of an obsolete national climate policy makes this enquiry even more challenging and opportune. For example, India formulated the National Climate Change Action Plan (NAPCC) in 2008 [25], wherein eight missions have been identified for mitigation and adaption actions, including for sustainable habitat, water, Himalayan ecosystem, green India and sustainable agriculture. The states are supposed to implement these keeping in view their own vulnerabilities and priorities. But in practice, this approach lacks knowledge on regional climate scenarios and their specific impacts. At the same time, the sectoral policies on environment, water, sanitation, energy and transport are not framed to specifically deal with climate change. With India urbanizing rapidly, cities need to become more responsive and resilient to climate impacts on five crucial urban sectors, namely water, infrastructure, buildings, urban planning and health. As per the local regulations (discussed in the governance section, energy is not a municipal subject, thus specific resilience related energy issues have been dealt within respective urban sectors, for e.g. infrastructure, buildings, etc. This investigation would thus serve to address these complex issues and challenges by testing how scientific knowledge on regional climate variabilities, theory and methods for adaptation measures and governance systems can be reasonably utilized to mainstream climate resilience in urban areas.

## 2. Review of literature

The process of implementing urban resilience theories is a complex and an evolving process. even more cumbersome in developing contexts, where lack of access to adequate, reliable infrastructure & urban services continues to impede the economic growth [26], while national environmental objectives and local governance issues are a key concern that not just undermine the climate cause, but associated direct, in-direct and ancillary benefits too [27]. Several studies in the last decade [28–32] have used diverse methods to demonstrate climate impacts on urban systems, owing to multiple exposures yet *there is no universal framework to analyze urban climate resilience*. According to Hammer et al. (2011) [33], there are three distinct approaches. The first is the *coping*/ *reduced sensitivity* or *engineering resilience* approach that emphasizes the importance of relatively short term means of enhancing strength, fortification and resistance of the critical infrastructure in urban systems [34–36]. Coping approaches bring immediate benefits that tend to diminish with new and diversified disasters and hence incur higher costs overtime. The second set of approach emphasize the *application of climate projections to determine future risks and the identification of specific measures* for responding to these [37–39]. The typical methodology follows prediction, prevention and making policies, practices and plans in order to avoid negative impacts of climate change [40]. In recent years, the Local Climate Zones system and its mapping emerged as an important approach to study the variations of local climates at the sub-national level [41]. Even though such a scientifically planned approach can help prevent losses and prepare for the changing climate, still adaptation cannot avoid all the impacts due to various limitations, which arise from socio-technical and governance related issues. Thus, a third set of framework argues complex urban systems *to strategically build ‘adaptive capacity’ in order to manage unanticipated stresses and shocks* [32, 42–44]. It closely aligns with the core idea of enhancing *climate resilience*, understood as the *ability of a system or society exposed to hazards to resist*, *absorb and recover from its effects in a timely and efficient manner*, *including through the preservation and restoration of its essential basic structures and functions* [45,46].

There is growing knowledge on the theory, methods, and practice of resilience across a variety of country and case contexts, that demonstrates how a resilience-based approach can help further improve infrastructure, vibrant societies, and sustainable environments and ecologies, among many others [47]. At the same time, it is pertinent to explore how pursuing urban resilience would be different from implementing climate adaptation measures in cities. Adaptation and resilience are two concepts originally developed in dissimilar problem contexts but which are of significant importance for our ability to respond to a changing climate. A better appreciation of the relationship between the concepts of adaptation and resilience will provide more effective tools to plan for, and respond to, current and future change [48]. This research makes an attempt in this direction as we focus on how could climate resilience be pursued in urban areas while considering adaptation alternatives, in addition to several other complex variables in climate geoscience and urban governance (discussed above).

Drawing from [23, 31, 48], we understand negotiated resilience from the perspective of incessant development policy and local governance based on localized climate change studies that offer clear benefits while employing downscaled Regional Concertation Pathways (RCPs) to establish a scientific justification for local adaptation policy.

However, in contrast to risk management, comprehensive approaches to assessing resilience at appropriate and operational scales, reconciling analytical complexity as needed with stakeholder needs and resources available, and ultimately creating actionable recommendations to enhance resilience are still evolving [49]. The 4×4 Resilience Matrix is one such framework for the performance assessment of integrated complex through an adverse event, where the rows describe the four general management domains of any complex system: physical, information, cognitive, social and the columns describe plan/prepare, absorb/withstand, recover, adapt [50]. Through case studies of cities, such tools have been used to organize the many government agencies and community institutions across different spatial scales that contribute to the operation of each critical service. In this research, we try to upscale similar tools for country-wide application in response to future climate scenarios, thereby arriving at entry points for mainstreaming resilience at various levels of governance, and incorporation of climate resilience building measures into different types of local/ urban plans.

## 3. Data and methods

**Fig 1:**
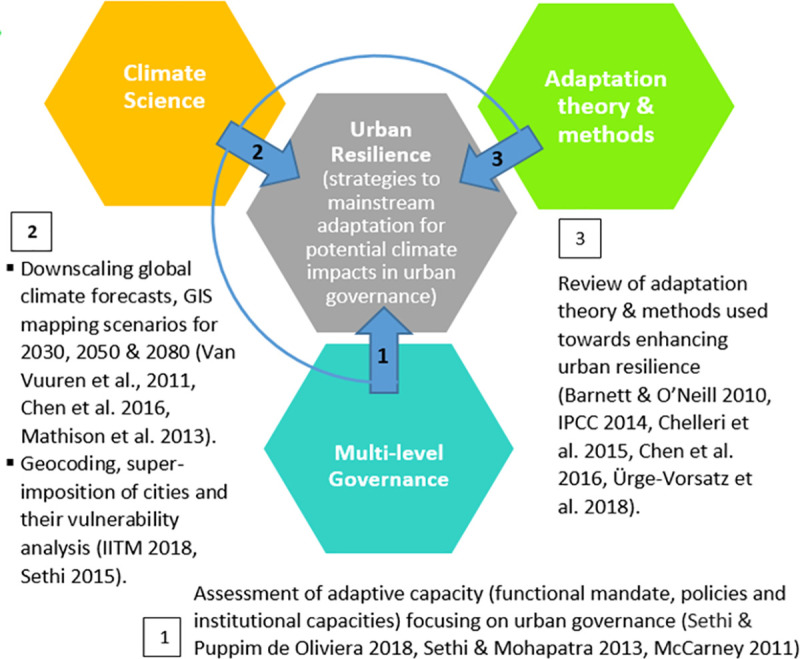
Theoretical framework for integrating climate science, adaptation theory and urban governance to deal with complex issues in urban resilience.

Based on the above theories, we formulate an integrated analytical framework (Fig 1), with the following techniques (and data) outlined to be performed sequentially:

### 3.1. Multi-level governance assessment

We draw from secondary sources of information spanning from 1992 to 2020, essentially in the form of policy studies and peer-reviewed papers, to conduct a qualitative assessment of multi-level governance systems, especially relevant to urban areas [10, 28, 51,52]. This assessment of the state of the affairs on the ground focuses on three core governance domains, namely functional mandate, plans and policies, and institutional capacities of Indian cities.

### 3.2. Simulating local climate scenarios

Using data from the Indian Institute of Tropical Meteorology (IITM) on their climate data portal [53], we retrieved the RCP 4.5 and 8.5 climate scenarios from the Coordinated Regional Downscaling Experiment (CORDEX. Here, we utilize techniques for downscaling global climate forecasts [54–56], mapping regional temperature (°C) and precipitation changes (in mm/day) across different geographic regions in Arc Map (version 10.2) for 2030, 2050 & 2080 and geocoding, super-imposition of cities for their vulnerability assessment [57]. The data sources and technique are elaborated in S1 Appendix.

### 3.3. Assessment of adaptation alternatives

On the basis of analyzing long-term forecasted climate scenarios and governance capacity of Indian cities, we identify adaptation options for definite climate impacts (temperature rise and extreme rainfall as the two primary ones, and sea-level rise and landslide as the secondary ones) for five key urban sectors in India, namely water, infrastructure, buildings, urban planning and health. In doing so, we refer to urban adaptation assessment and prioritization methods [3, 58–60]. We evaluate these on the basis of (1) priority (medium, high, very high), (2) implementation time (short-, mid- and long-term) and (3) intervention level (State, city, sub-city, building, etc.).

### 3.4. Negotiating resilience into governance

Based on definite adaptation measures in key urban sectors, we infer specific policy actions and governance mechanisms to be undertaken by different stakeholders at multiple scales. We expand matrix methods used in individual cities [50] to infer negotiated climate resilience measures for national, state and local levels of governance, particularly focusing on their internalization into different types of urban policies and plans.

## 4. Discussion of results

### 4.1. Multi-level governance assessment in India

The multi-level governance of environment and climate identifies the gaps and needs in the functional mandates, plans and policies, and the institutional capacities of Indian cities, as elaborated:

#### 4.1.1. Functional mandate

Climate actions are concerned with multiple-tiers of government–national, state and the local. The nodal institution governing climate change at the national level is the central ministries, notably the Ministry of Environment, Forest and Climate Change (MoEFCC) while the states have similar departments with differing nomenclature such as Department of Environment and Forest (in Assam), Department of Forest and Wildlife Preservation (Punjab); Forest, Ecology and Environment Department (Karnataka). The MoEFCC is responsible for planning, promotion, co-ordination and implementation of India’s climate policies and programmes. It has framed the overarching NAPCC under which respective state governments are implementing their State Action Plan for Climate Change (SAPCCs). The SAPCCs address a range of concerns including, assessing community vulnerabilities, identifying climate change effects across states, understanding the impact of economic activities on the environment, and proposing strategies to respond to climate change [61]. However, it is observed that the states continue to prioritize protection of natural environment, forests and wildlife over climate concerns. For instance, the vision statement of the Karnataka’s environment department is “Conservation, management and development of forests and tree growth, on sustainable basis, for present and future generations” [62]. Climate change has not yet become a major focus area for these departments. The governmental actions in dealing with climate change are chiefly driven by climate mitigation, with adaptation being a co-benefit [10, 63].

Likewise, for urban areas, the Ministry of Housing and Urban Affairs (MoHUA), is the nodal ministry and implements the National Mission for Sustainable Habitat. The MoHUA deals with development, water supply, sanitation, transport, housing, and poverty alleviation in urban areas, which will be severely impacted by global warming. However, climate change has not been integrated into the urban policies and programmes of MoHUA, except for urban flooding, wherein it recently prepared guidelines to deal with increasing flood threat in urban areas. Similar to the state environmental departments, exists a parallel vertical apparatus of urban departments in respective states. While higher levels of government formulate policies, programmes and guidelines, their implementation is done by the concerned state line departments and urban local body (ULB). A ULB can be a municipal corporation, municipal council, town committee, and in certain cases a development authority. The normative functions to be performed by a ULB are provided in the 12^th^ Schedule of the 74^th^ Constitution Amendment Act [64]. The functions cover urban planning including town planning, regulation of land use, planning for economic and social development, water supply, public health, urban forestry, protection of environment and promotion of ecological aspects, slum improvement, urban poverty alleviation, provision of parks and gardens, among others [51, 65], a notable exception being energy needs/ electricity. The modes by which these are planned and implemented is discussed in #4.2 Polices and plans. Thus, there is not just a noticeable gap in clearly spelling out climate resilience in the legal provisions applicable to ULBs, but also in fully empowering them to deal with the imminent challenge on the ground.

#### 4.1.2. Policies and plans

Currently, India is implementing several urban programmes in mission mode [66] like the Smart Cities Mission (SCM), Atal Mission for Rejuvenation and Urban Transformation (AMRUT), Swachh Bharat Mission (SBM). There are capacity building components designated under these urban missions, which mainly focus on improving infrastructure and functioning of cities, and the use of new technologies to improve efficiency and provide a better quality of life in urban areas. However, building technical capacities to generate regional climate change projections that accurately assess climate vulnerability and impacts in cities and mitigate those has not been incorporated in these initiatives (see Table 1).

**Table 1:** The gaps in climate resilience components in government’s urban missions.

Mission / Initiative	Infrastructure	Gaps in Climate resilience
**Smart City**	Water supply, sanitation and waste management, urban mobility and public transport, health and education, electricity, affordable housing, robust IT and digitization	Lacks assessment of urban climate vulnerability, future impacts and resilience measures in defining smart city features, strategy and proposal preparation guidelines crucial to planning, infrastructure, buildings, health, etc.
**AMRUT**	Water supply, sewerage facilities and septage management, storm water drains, green spaces, parks and recreation centres, parking spaces and pedestrians non-motorized public transport	Provision of open spaces and transportation facilities lacks resistance to floods, heat, sea-level rise, landslides, etc. The refurbishment of old infrastructure and creation of new one lacks strengthening/ coping mechanisms to endure foreseeable climate impacts. The water supply, drainage and sewerage management infrastructure lack mandatory reforms provision for water recycling and harvesting.
**HRIDAY**	Sanitation toilets, drinking water facilities, solid waste management, traffic management, street furniture, public transport and parking	Creation of new facilities lack pre-audits for engineering resilience against future climate impacts.
**Housing for ALL (Urban)**	Civic amenities and infrastructure	There is no mandatory provision to protect buildings and civic infrastructure against climate induced natural disasters
**Swachh Bharat Mission**	Urban households toilets, community toilets, public toilets and urinals, solid waste management	Creation of new facilities lack pre-audits for engineering resilience against future climate impacts.

Source: [66]

The ULBs execute functions in the 12^th^ Schedule through preparation and implementation of multiple city-wide plans, including Town Plan/ Physical Land use plans, Storm water management plan, Transportation Plan, Local Economic Development Plan or Strategy, Informal Settlement Upgradation Plans, Sites and Services Plan, Slum Development Schemes, Solid Waste Management Plan, Energy Management Plan, Water Management Plan, Sewer/ Liquid Waste Management Plan, Emergency Management Plan, Public Health Plan. Although these functions have considerable influence on mitigating disasters and climate resilience of a city, in practice, many states are still in the process of actually devolving functions such as urban planning, regulation of land use, and urban forestry to the concerned ULBs. In addition to the governments, during the last decade, private and non-governmental organizations such as Local Governments for Sustainability (ICLEI), TARU Leading Edge, The Energy and Resources Institute (under the Asian Cities Climate Change Resilience Network funded by the Rockefeller Foundation) and the German Society for International Cooperation (GIZ) have initiated pilot projects to create awareness and build capacity of urban governments on climate change issues, but their involvement is confined to select cities of Gorakhpur, Surat, Indore, Guwahati, Shimla, Bhubaneswar, Mysore [67]. The climate initiatives need a significant scaling up and shift in focus from mitigation to resilience action.

#### 4.1.3. Institutional capacity

The institutional capacities include an assessment of both technical and financial conditions, particularly those on the ground. Technically, India’s Nationally Determined Contribution (NDC) document submitted to the UNFCCC claims that smart solutions like recycling and reuse of waste, use of renewables, protection of sensitive natural environment will be incorporated to make cities climate resilient [68]. In practice, there is a lack of capacity at multiple levels of the institutional apparatus to address environment and development issues. For instance, the 74th CAA provides a framework to enable participation of citizens in urban governance through formulation of wards committees in cities, yet only handful of states constituted these [69]. The subject of ‘climate change’ being excluded in both the 12^th^ schedule (list of ULB functions) and the ongoing urban policies and programmes, there is little institutional attention on creating adequate hard climate infrastructure and equipment or trained technical and managerial staff for building resilience, be it in the state line departments or the ULBs. Merely adding climate change to their existing organizational mandate without provision of additional staff and funds poses a range of governing challenges [70]. In addition, technical capacities on urban resilience need to be built for all local stakeholders including the public representatives, private sector, non-governmental organizations, civil society organizations and the community at large. For instance, Surat established the Urban Health Climate Resilience Centre (UHCRC) and Surat Climate Change Trust (SCCT) as umbrella institutions [71] to organize all stakeholders and concerned departments on climate agenda.

There is a lengthy discourse on the financial condition of Indian cities. The availability of funds to finance climate projects remains an unceasing challenge in Indian cities [72]. The ULBs who have to take decisions and governance measures, are financially challenged and lack adequate resources to even carry out normal functions properly [73]. India’s annual budgetary investments in urban sector is about 1.5–1.7 per cent of the country’s GDP compared with the Asia’s average of 5.7 per cent, grossly falling short of requirements and explains the persistence of huge infrastructure shortages across cities and towns despite infrastructure development being central to mission programmes such as the Smart Cities and AMRUT [74]. Public financing continues to be the principal mode of funding urban infrastructure with limited role of municipal bonds, land value capture and private sector investments. The constraints imposed by weaknesses in existing urban public finance institutions complicates envisioning the smooth pooling of blended finance from multiple sources across multiple scales [75]. Nevertheless, there is a discernible gap in: (a) integration of climate resilience provisions in national urban mission programmes that can assist funding adaptation oriented local development projects, (b) routing of urban resilience investments from district disaster mitigation funds, green cess in environment and energy, and the penalties imposed on environmental violations by the National Green Tribunal and the Supreme Court of India, (c) sourcing of non-governmental funds from private sector, land monetization and infrastructure bonds

### 4.2. Simulating local climate scenarios

The simulating (downscaling & mapping) climate scenarios involves downscaling of global climate models for two Representative Concentration Pathway (RCP): RCP 8.5 and RCP 4.5 over three timelines: short-term (2030s), medium-term (2050s) and long-term (2080s). The resulting six temperature and precipitation anomaly scenarios are mapped and super-imposed with urban geo-database of cities (location- latitude, longitude) for all regions/ states of India to analyze local urban vulnerabilities, a summary of results follows:

#### 4.2.1. Temperature anomalies

**Fig 2:**
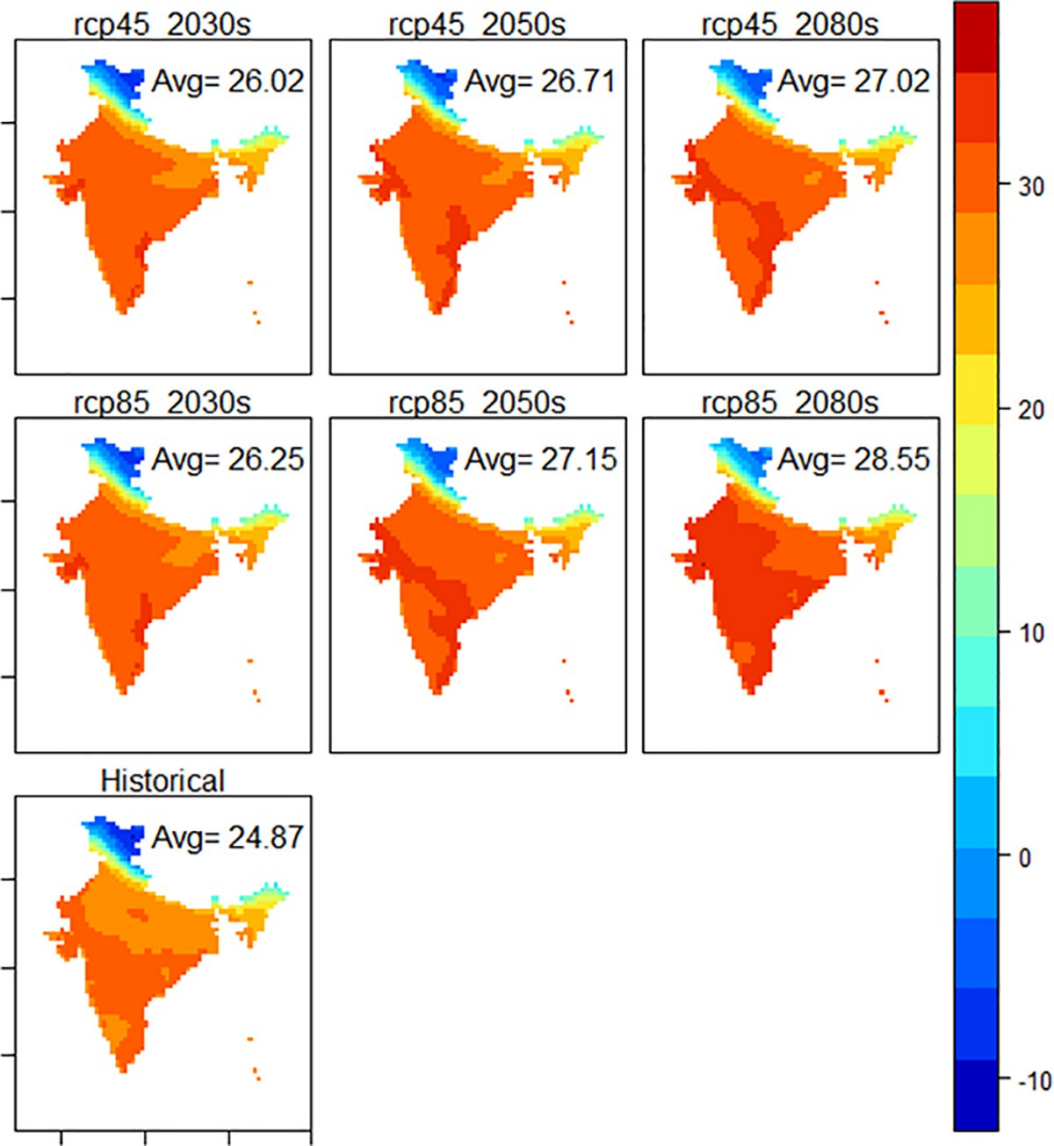
Distribution of maximum near surface air temperature under different RCPs during different time-periods against historical reference. Source: World Climate Research Programme and PLOS ONE under CC BY 4.0 license [80].

The spatial distribution of future anomalies in maximum mean surface temperature in the country ranges from 1.15 (in 2030) to 4.68 degC (in 2080) and is very similar to patterns of historical average temperature (see Fig 2). The detailed temperature anomaly maps for different regions/ states are provided in S2 Appendix. In 2030s, the *Western Himalayas* particularly north western parts of Jammu & Kashmir will experience increase of 2.3°C under RCP 8.5. In 2050s, the increase could be as high as 6°C. In *Eastern Himalayas*, the temperature rise could range from 1.42°C in 2030s under RCP 4.5 to 4.48°C in 2080s under RCP 8.5. The temperature increase is also high in dry and semi-dry regions covering *plains and hills of Gujarat* and *western dry regions* of *Rajasthan*. The change in *western dry Rajasthan* is predicted to range from 1.63 to 4.6°C in 2080s and 1.57°C to 4.46°C in *Gujarat plains and hills* under RCP 8.5. The *Central plateaus and hills*, may experience an increase of 2.55°C in the maximum temperature by 2080s under RCP 4.5, while the increase could be 4.44°C under RCP 8.5. The drought prone region has important cities like Bhopal and Gwalior from Madhya Pradesh, Jaipur, Kota, Ajmer and Udaipur from Rajasthan and Jhansi from Bundelkhand. The *Upper Gangetic Plains* (UGP) and *Trans Gangetic Plains* (TGP) that inhabit a large number of Class-I cities (at least 100,000 population), are also predicted to experience high increase in maximum temperatures: 1.23°C to 2.13°C for *UGP* and 1.39°C to 2.36°C for *TGP* under RCP 4.5 from 2030s to 2080s. Under RCP8.5, the situation could even be worse with maximum increase of 2.13°C for *UGP* and 3.62°C for *TGP*.

Meanwhile, a significant variation in the minimum near surface air temperatures in the country is expected to be in *Western Himalayas*. The anomaly could be as low as 2.47°C in 2030s under RCP4.5 or as high as 7.10°C in 2080s under RCP8.5. The *Eastern Himalayas* are expected to have maximum increase of 3.58°C in 2080s under RCP4.5 and 5.77°C under RCP8.5. An increase of 1.74°C is predicted for *western dry region* in 2030s under RCP4.5 and 2.92°C in 2080s. This minimum increase will be higher under RCP8.5, which is expected to be 1.93°C, 3.11°C and 4.99°C in 2030s, 2050s and 2080s. Another region that is threatened due to increase in minimum temperatures is *TGP*, which expects an increase of 1.52°C in 2030s, 2.32°C in 2050s, and 2.63°C in 2080s under RCP4.5. The corresponding increase under RCP8.5 would be 1.73°C, 2.82°C and 4.56°C.

#### 4.2.2. Precipitation anomalies

**Fig 3:**
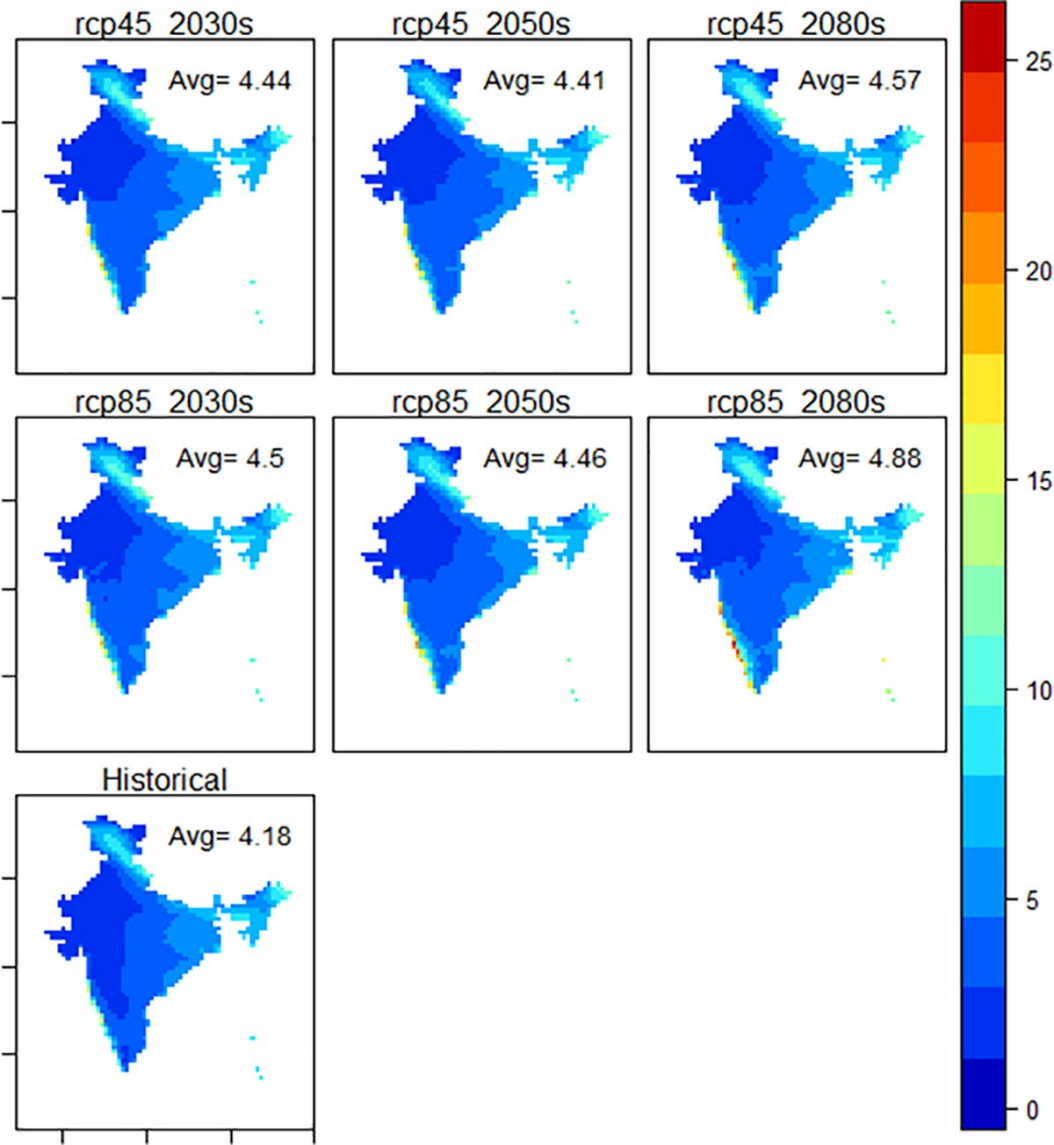
Distribution of precipitation (mm/day) under different RCPs during different time-periods against historical reference. Source: World Climate Research Programme and PLOS ONE under CC BY 4.0 license [80].

The precipitation anomalies vary substantially from 0.23–0.70 mm/day across the country (see Fig 3). The detailed rainfall anomaly maps for different regions/ states are provided in S2 Appendix. The rainfall is expected to increase along the *western coast plains and hills*, and on the contrary, the *western Himalayan* region is expected to become drier over the years. The *northeastern states* are expected to experience significant variability in precipitation, ranging from -0.80 mm/day (2050s, RCP8.5) and could be as high as +1.5 mm/day under (2080s, for both RCP8.5 and RCP8.5). An increase in precipitation is expected in *western coastal plains and hills* that covers coastal parts of Karnataka, Kerala and Maharashtra including 25 cities, some of which are Mangalore, Thiruvananthapuram, Kochi, Kozhikode, Kollam, Thrissur, Alappuzha, Palakkad, Mumbai and their surrounding suburbs. The increase in precipitation in the region during 2030s is predicted to be 4.66 mm/day under RCP 4.5 and 3.98 mm/day under RCP 8.5. The greatest increase in precipitation is expected in 2080s, as high as 6.4 mm/day under RCP 4.5 and 11.35 mm/day under RCP 8.5. Port Blair on the Andaman & Nicobar Islands is expected to experience an increase of 1.81 to 3.13 mm/day in average precipitation rate in 2080s under RCP 4.5. However, this change might vary between 3.13 and 6.75 mm/day under RCP 8.5.

### 4.3. Assessment of adaptation alternatives

Based on different temperature and precipitation anomalies, five possible pan-India situations emerge (Table 2) for influencing regional vulnerabilities and climate impacts. Out of these, Situation 4 (decrease in projected temperature, decrease in projected precipitation) and Situation 5 (decrease in projected temperature, increase in projected precipitation) have not been observed.

**Table 2:** Multiple situations of projected temperature and precipitation across Indian states/ regions.

Situation	Projected temperature	Projected precipitation	Observation	Region/ State (refer Annex 2)	Possible adaptation alternatives
1	Increase	Increase	√	Andhra Pradesh & Telangana, Chattisgarh (North), Himachal Pradesh, Jammu and Kashmir, Karnataka, Kerala, Maharashtra & Goa, Tamil Nadu & Puducherry, Uttarakhand	Discussed in section 5.1 (extreme temperature, drought, heat waves) & 5.2 (extreme rainfall, floods)
					Section 5.3 in particular for hill areas of Himachal Pradesh, Jammu and Kashmir
					Section 5.3 in particular for coasts of Andhra Pradesh, Karnataka, Kerala, Maharashtra & Goa, Tamil Nadu & Puducherry
2	Increase	Decrease	√	Jharkhand	Discussed in section 5.1
3	Decrease	Decrease	X	None observed	-
4	Decrease	Increase	X	None observed	-
5	Increase	Variable/ uncertain trends	√	North-East & Sikkim, Bihar, Chattisgarh (South), Gujarat, Haryana, Chandigarh and Delhi, Madhya Pradesh, Odisha, Punjab, Rajasthan, Uttar Pradesh, West Bengal	Discussed in section 5.1 & 5.2
					Section 5.3 in particular for coasts of Gujarat, Odisha, West Bengal

For details of increase & decrease of projected temperature and precipitation in each state/ region, refer S2 Appendix.

**Fig 4:**
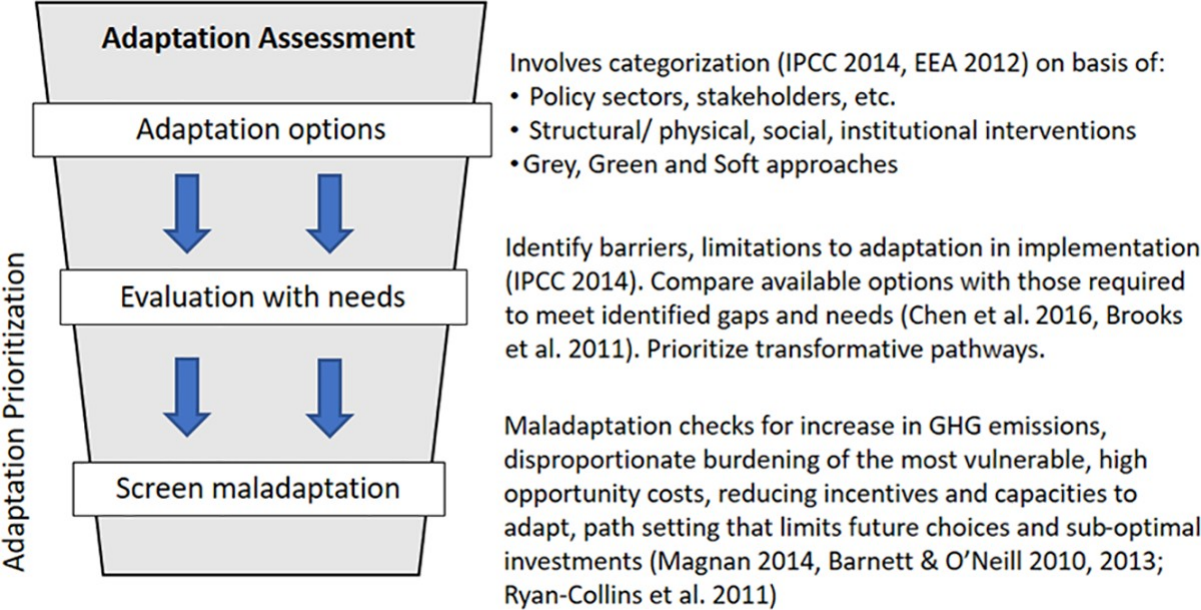
Assessment of adaptation alternatives.

Theoretically, there are numerous climate adaptation alternatives and these need to be methodically considered and prioritized (see Fig 4). The primary aim of the adaptation prioritization is assessment of the options against local needs while avoiding maladaptive practices [76]. During adaptation assessment, all the available alternatives can be listed on the basis of *sectors*, *involved stakeholders* or the *type of intervention* [3]. The prioritization selects ‘*transformative’* options over just coping mechanisms and excludes low-priority alternatives and ‘*maladaptation’* practices. Maladaptation is defined as a process that results in increased vulnerability to climate variability and change, directly or indirectly, and/or significantly undermines capacities or opportunities for present and future adaptation [58, 77, 78]. For instance, the use of air conditioners for cooling in very hot weather can result in enhanced emission of GHGs. During prioritization, our main focus has been to minimizing vulnerability to potential climate impacts and improve city resilience. The resulting adaptation alternatives are reported for temperature rise, heat waves and droughts (S3 Appendix), extreme rainfall and flash floods (S4 Appendix), landslides (S5 Appendix) and sea-level rise (S6 Appendix). We evaluate these on the basis of (1) priority (medium, high, very high), (2) implementation time (short-, mid- and long-term) and (3) intervention level (State, city, sub-city, building, etc.).

Upon assessment, we found that crucial adaptation measures for temperature rise, heat waves and droughts include conjunctive water management (the coordinated use of both groundwater and surface water in order to maximize sufficient yield), rainwater harvesting, water and energy demand management, strengthening of electricity distribution system, exploring renewable sources such as solar power and biomass, adoption of cool/green roofs, green buildings rating systems; urban greens, cool pavements and tree-lined streets for mitigating the UHI effect. In addition, an early warning system for heat waves and decentralizing health facilities to provide easy and timely access to health services to the people will create necessary preparedness and resilience. Measures for heavy rainfall and flash floods include improving and maintenance of water conveyance systems, drains, flood gates, dikes, bulkheads, culverts, bridges, power installations. In buildings, stilts, elevated entrances, green roofs, provision for run-off storage will reduce flood impacts. New cities and revision of Master Plans in existing cities can incorporate ‘sponge cities’ and ‘urban farming’ features to reduce runoff intensity, peak flow and maintain water balance. To reduce loss to property and life, early warning system, disease surveillance system, primary health centres and mobile clinics can be used to serve slums and low income habitation as these localities are likely to be worst affected. As urban resilience measures, hill and coastal cities should use risk hazard maps for rational land use planning and decision-making. Safety screening studies should be carried out to identify vulnerable slopes for strengthening through natural vegetation and structural measures.

## 5. Conclusion and recommendations

### 5.1. Negotiating climate resilience in local planning and governance

Unlike developed cities that are more amenable to opting transformative resilience strategies, Indian cities are in a developing process and need to focus on enhancing adaptive capacities while enormously expanding their infrastructure. Developing infrastructure and services that also meet climate requirements will build long-term resilience by reducing the vulnerability and gradually boosting adaptation to the changing climate. Based on the urban governance needs, climate scenarios (future temperature and precipitation changes) and identified adaptation measures, we devise urban resilience strategies for the impending climate impacts. These need to be negotiated through policy actions and governance mechanisms by different institutions and stakeholders at multiple levels (Table 3). The federal structure of policy-planning and implementation in India makes a potential case for climate resilience by integrating adaptation at all the three entry points- national, state and local urban levels of governance.

**Table 3:** Entry points for negotiating resilience at various levels of governance.

	Policies	Institutions and stakeholders
**National Level**	Climate: National Missions as part of the National Action Plan on Climate Change (upon updating)	Ministry of Housing & Urban Development, Ministry of Environment, Forests and Climate Change, Parliament’s Standing committee on urban development, National Institute of Urban Affairs, other national level research and policy institutes
	Urban: Smart Cities Mission (SCM), Atal Mission for Rejuvenation and Urban Transformation (AMRUT), Swachh Bharat Mission (SBM).	
	Sectoral policies in water, transport, buildings, energy, etc.	
**Sub-national/ State Level**	State Agendas and Action Plans on Climate Change	Regional Centres for Urban and Environmental Studies, State line departments, State line departments/ ministries of land, urban development and environment. State relevant research institutes and universities
	Sectoral Policies	
	State Five Year Plans	
**City Level**	Master Plans	Urban local bodies (municipal organizations), development authorities, departments concerned with land, housing, slums, transport, energy, water, public health, storm water, sewerage, environment, solid waste, etc. Local educational institutions, private sector, philanthropies and non-governmental organizations.
	City Development Plans	
	Disaster Management and Resilience Plans	
	City Mobility Plans	
	City Sanitation Plans	

This necessitates appropriate provisions to be incorporated into the Town and Country Planning Act and zoning regulations, development control rules and building bye-laws, district planning manuals, National Building Code, Urban and Regional Development Plan Formulation and Implementation Guidelines [79]. Most importantly, resilience measures are to be integrated in different types of city policies/plans (Table 4), in addition to their general purpose that ULBs are mandated to frame and execute, particularly master plans, plans related to landuse, water, transport, housing, public health and waste, among others.

**Table 4:** Incorporation of climate resilience building measures into different types of local/ urban plans.

Type of Plan	General Purpose	Climate resilience building measures
Town Plan/ Physical Land use plans	• Identifies development hazard areas (steep slopes, flood plains, etc.)	• Highlight “hot spots” or “no development areas” where climate change impacts are likely to be most severe
	• Identifies areas (zones) for different types of development (i.e., housing, commercial, industrial, etc.)	• Set policy direction on “climate friendly” and/ or “climate resilient” infrastructure and services for e.g. storm water management, water, etc.
	• Provides long-term direction on land use and development, work centers, transportation and overall community development	
Storm water management plan	• Improves storm water management, including drainage infrastructure	• Zonation of flood hazard areas and marking of high flood lines in habitations.
		• Climate proofing of storm water structures, conveyance, drains, pumps, etc.
		• Mandatory provisions for flood and coastal management, including appropriate sustainable defences.
		• Increase permeability of paved areas and ground water recharge in both drought and flood prone areas.
		• Directing new infrastructure to “safer” areas not as risky to climate change impacts (i.e. can attract or pull development to serviced areas).
Transportation Plan	• Improves mobility and infrastructure for motorized and non-motorized transport	• Identify and strengthen “weak links” in transport networks e.g. rail-roads, highways, bridges, culverts, pathways, threatened by storm surges, flooding, etc.
		• Identify and designate emergency routes
		• Improving road infrastructure and modes commonly used by the urban poor and vulnerable groups
		• Integrate resilience efforts with mitigation funds aiming at prioritizing non-motorized transportation, reduced congestion and carbon emissions.
Local Economic Development Plan or Strategy	• Identifies and prioritizes economic activities and livelihoods (i.e., jobs, capacity, infrastructure, etc.)	• Reduce urban poverty levels for key vulnerable groups e.g., women, children, urban poor.
		• Promote “green development” and/ or “climate friendly” opportunities
Informal Settlement Upgradation Plans, Slum Development Schemes	• Develops policies and plans to improve services, infrastructure and sanitation in informal settlements and slums	• Identify risks to local people, hutments, plinths, alleys, slope failures) and respond through transformative measures like infrastructure improvement
		• Identify residential clusters in high hazard areas and/ or develop relocation strategy or planned-retreat.
		• Capacity development/ training of stakeholders and regular monitoring of efforts.
Solid Waste Management Plan	• Improves solid waste management, including collection, transportation and disposal infrastructure	• Align with mitigation efforts through improved materials recycling and/or reuse
		• Promote scientific landfills to mitigate leaching, inundation, air pollution and GHGs
Water Supply Management Plan	• Improves water supply, management and distribution	• Augment water reserves for extreme arid months and strengthen municipal supply, treatment and distribution capacities
	• Improves water conservation	
		• Accelerate water conservation and water demand strategies and to manage and adapt to potential shortages
Sewer / Liquid Waste Management Plan	• Improves waste water/ sewer management	• Develop guidelines for “climate proofing” infrastructure (i.e., build and locate infrastructure to withstand and function during extreme climate events)
		• Identify and prioritizes high risk areas where new facilities are most needed to reduce climate change impacts amongst vulnerable groups
		• Identify options to reuse wastewater (grey water) for urban agriculture and horticulture
Energy Management Plan	• Improves energy generation options, distribution, and conservation	• Strengthen energy generation and distribution systems and infrastructure
		• Support green energy, decentralized supply and energy storage facility to counter black-outs and brown-outs
Emergency Management Plan	• Improves disaster response preparedness	• Identify climate change disaster risks, and adaptive capacity.
	• Identifies ‘hot spots’ (i.e., areas and groups vulnerable to disaster)	• Support and facilitate infrastructure and planning to reduce climate change-related disaster impacts
Public Health Plan	• Focuses on disease prevention and public safety improvements	• Prioritize health risks e.g., disease, accident, etc. associated with climate induced disasters.
		• Support and facilitate infrastructure and planning improvements in public-health systems to minimize and manage heat stress and outbreak of vector borne diseases.

The building of resilience into urban governance for heat waves, extreme rainfall events, sea level rise, and landslides would invariably span through regulation, provisioning/ enabling and voluntary mechanisms. Capacity building of officials and other stakeholders at all levels- national, state and local needs to be undertaken on climate resilience in government’s urban policies, programmes and projects, as mandatory reforms linked to financial grants. In addition to training, regular monitoring of mechanisms within these policies/ plans will help officers analyze resilience actions, evaluate their progress and impacts to the society.

The recommendations for making resilient cities include multiple but specific initiatives embracing structural, green and regulatory measures cutting across diverse policy sectors. For instance, for heat waves cities can opt for cool/ green roofs, implement conjunctive water management, regulate groundwater extraction, use solar energy where possible, switch to LED lighting, design neighbourhoods to allow air flow without obstruction, regulate housing densities, decentralize health facilities and make them accessible to all. Likewise, for heavy rainfall and flooding, improve drainage network, remove encroachment on drains, build houses on stilts where required, elevate entrances to buildings, harvest rainwater to reduce peak flow, provide adequate open and green spaces to absorb water and reduce runoff. For systematic internalization of these into urban policies, cities should prepare City Resilience Strategy documents, set up new institutions or departments in local bodies to deal with climate change, and educate and involve people in the managing climate impacts.

Negotiating climate governance also requires reliable financial and monitoring mechanisms to be put in place. In absence of any designated and reliable climate financial framework at national and state level, governments need to commit special funds for urban resilience activities in ongoing government programmes like NAPCC, SCM, AMRUT, SBM, etc. The funds would be linked to measureable outcomes in state programmes under SAPCC and that of the line departments in urban, water, health, energy, sewerage sectors. However, at the local level, funding for climate resilience has to be mobilized through state grants, their annual budgets and planned projects. As an example of a case where climate change has been effectively internalized is the city of Surat where the municipal corporation has added a new head of ‘Climate Change’ into their municipal budget.

### 5.2. Research and policy imperatives

With complex and disparate knowledge on future climate scenarios, adaptation measures and governance capacities at the local level in developing countries, this research started with a focused enquiry into how cities would respond to imminent temperature and precipitation changes? We conclude this investigation with some interesting and novel research outcomes-

1. With marked technical, functional and governance complexities in the Indian case (including absence of a national urban policy and outdated NAPCC/SAPCCs), this research demonstrates an innovative methodology by which urban resilience can be directly pursued by downscaling global RCP forecasts to map long-term temperature and precipitation scenarios, evaluating regional climate vulnerabilities, detailed assessment of adaptation measures at the sub-national level, followed by mainstreaming resilience strategies into the existing urban policies and governance.
2. The temperature and precipitation forecasts collectively suggest escalations in the intensity of climate events like heat waves, urban flooding, landslides, sea level rise with most cities being impacted by more than one hazard. Their regional geographical location would determine which hazards will be significant, for instance coastal cities face multiple threats of cyclone, extreme precipitation and sea level rise. The northern plain cities experience heat waves and events of extreme rainfall during monsoons; while cities in the hills are prone to heavy precipitation related landslides. We present our findings as state level maps that are useful to prepare tailor-made resilience measures for their respective cities.
3. The urban areas are highly exposed to the rapidly changing climate although their functional and institutional capacities in developing countries are disproportionately limited. The case of India shows how climate mitigation is prioritized at the national level, while state environment departments customarily deal with forestry and environmental pollution. This methodology, actively highlights several untapped resilience building issues that have potential co-benefits with urban planning, water, building, energy and health related policies/ plans at multiple levels, and most importantly at the city level.
4. In spite of its usefulness, the methodology may offer certain limitations during application in the developing contexts. It is a dynamic milieu facing an ever evolving technology, society and environmental data. The scientific projections available for the most current state of the knowledge and with advances in climate scenarios and geo-spatial mapping, these would tend to become rather precise in future leading to improved micro-planning interventions. The technological progress and expanding markets in developing countries too would provide more high-tech resilience measures like water saving technologies, green roofs, thermal insulation, water-proofing in buildings, etc. while it is unpredictable to ascertain how the people would respond to new ideas and technologies.
5. Thus, it is vital that the selection of solutions by individual cities will be based on their specific experience to disasters, current and potential hazard, governing capacity, investment requirements and the expected level of resilience it would provide. Once again, the financial feasibility of climate resilience measures is an externality in the present methodology as this would be project and location specific. A cost-benefit analysis will need to be done for different options before selecting the most suitable solution at the city or regional level.

Despite of mixed outcomes, the theoretical/conceptual contribution of the paper to the literature on urban climate resilience cannot be understated. In absence of a universal framework to analyze urban climate resilience, there is a preponderance of studies tending to focus too much on either of the disciplinary knowledge i.e. national climate forecasting, coping/ engineering resilience mechanisms, or appraisal of local governing capacities. On one hand this creates specialized knowledge, it becomes utterly complex while applying it on the ground. In contrast to this results-based and statist construct of urban climate resilience, this investigation advances the thought of negotiated resilience as a process-based approach. It demonstrates how this negotiation can be systematically pursued between the inter-disciplines (of climate science, multilevel governance, adaptation) by first grounding the need of urban resilience through multi-level governance framework, then downscaling regional climate scenarios at an appropriate scale where adaptation alternatives could be assessed to integrate resilience building actions within the prevailing policy practice. The research bears several practical implications, both for the case study of India and for widespread global applications, particularly in developing countries. We recommend to actuate climate resilience measures in the national and sub-national policy practice (in India) and suggest corresponding imperatives for international cases, academics and decision makers (Table 5).

**Table 5:** Recommendations to actuate climate resilience measures in practice, academics and decision making.

	Recommendations to steer climate resilience measures in policy practice	Imperatives for international cases, academics and decision makers
**National Level**	(1) Revision of the 12th Schedule of the 74th Constitutional Amendment and delegation of powers related to climate subject to ULBs,	(1) The parliaments should delegate legal and executive powers for climate action to the municipalities/ urban bodies
	(2) Inclusion of climate resilience into Smart Cities Mission, AMRUT, HRIDAY, Swachh Bharat and Housing for All and other sectoral plans	(2) Inclusion of climate resilience measures into flagship development programmes of the national government
	(3) Updating of Directives on civil works and procurement in central/ state governments, public sector undertakings, defence organizations, financial institutions, etc.	(3) Revision of civil works, procurement/ tendering procedures in central government agencies to incorporate climate resilience
		(4) A national commission of town planners, architects, academics, etc. should be involved in formulating climate resilient development strategy, revision of urban/ regional planning norms, guidelines, landuse zoning and building codes
	(4) Revisiting Urban and Regional Development Plan Formulation and Implementation, (URDPFI) Guidelines, the National Building Code and local building bye-laws in states and cities	
**State Level**	(5) Mandatory inclusion of GRIHA guidelines into commercial, industrial and large scale residential buildings	(5) The provincial governments should make implementation of green building guidelines compulsory in their cities
	(6) Convergence of resilience building measures with sectoral plans of the state governments	(6) Multiple stakeholder groups should be involved to explore synergies of resilience measures within the sectoral plans in the province
	(7) Climate resilient urban policies of respective state governments, followed by master plans of development authorities, municipal bodies in their jurisdiction	
		(7) State governments should institute expert committees to include climate resilience in local policies and master plan framework
**City Level**	(8) Indian cities need to prepare City Resilience Strategy document that would contain present status of infrastructure, services and institutional arrangements, climate change projections for the near and long term, and actions to be taken in different sectors that will make cities climate resilient.	(8) Multiple stakeholders including government agencies, expert groups, academics and local representatives/ people should participate in formulating a climate resilience strategy for their cities. They can come together to form an umbrella body housed within the municipality, that practically serves as the nerve centre for urban resilience, community preparedness and disaster recovery too.
	(9) On the lines of UHCRC and SCCT, Indian ULBs should host institutions to strengthen climate resilience. Such an arrangement can improve understanding, cooperation, and coordination amongst stakeholders, with urban local body being a major stakeholder.	
		(9) Regular monitoring mechanisms built into municipal actions would ensure greater efficiency and timeliness in climate planning & governance.

At the urban scale, we suggest that cities need to prepare City Resilience Strategy document that would contain present status of infrastructure, services and institutional arrangements, climate change projections for the near and long term, and actions to be taken in different sectors that will make cities climate resilient. Such an arrangement can improve understanding, cooperation, and coordination amongst stakeholders, with urban local body being a major stakeholder. Knowledge sharing on how certain resilience measures have benefitted one city will help others to consider newer options that may be more efficient and effective. It is a low-cost but high impact alternative in developing contexts to reduce human and material losses and better implement local actions. Last but not least, regular checks and monitoring mechanisms built into municipal actions would ensure greater efficiency and timeliness in climate governance at the local level.

## Supporting information

S1 Appendix Description of method. (DOCX)

S2 Appendix Forecasted climate variability in temperature (in °C) and precipitation (in mm/day) against historic trends across different states/ regions of India. (DOCX)

S3 Appendix Adaptation for temperature rise, heat waves and droughts. (DOCX)

S4 Appendix Adaptation for heavy rainfall events and flash floods. (DOCX)

S5 Appendix Adaptation for landslides. (DOCX)

S6 Appendix Adaptation for sea level rise. (DOCX)
