# Supplementary material for: How to tackle complexity in urban climate resilience? Negotiating climate science, adaptation and multi-level governance in India
Source: PLoS One. 2021 Jul 1;16(7):e0253904. doi: 10.1371/journal.pone.0253904 (PMC8248603; doi:10.1371/journal.pone.0253904)
Supplement: S1 Appendix — (DOCX) [file pone.0253904.s001.docx]

**S1 Appendix. Description of Method**

The method to simulate local climate scenarios adopts three analytical techniques: (1.1) Downscaling forecasted regional climate scenarios, (1.2) Mapping the temperature and precipitation changes, and (1.3) Superimposing the geo-locations of major urban settlements

**1.1. Downscaling forecasted regional climate scenarios**

The AR5 report (IPCC 2014) has proposed four climate change scenarios based on four different Representative Concentration Pathways (RCPs) viz. RCP 2.6, RCP 4.5, RCP 6.0 and RCP 8.5. RCPs are four different possible climate futures depending upon varying greenhouse gas (GHG) concentration trajectories or pathways and most commonly used for climate research and modelling. These RCPs have been named after a possible range of radiative forcing^[[1]](#footnote-1)^ values in the year 2100 relative to pre-industrial values (+2.6, +4.5, +6.0, and +8.5 W/m^2^, respectively). These scenarios take into account a time series of emissions and concentrations of GHGs and aerosols and chemically active gases, as well as land use/land cover. RCP 2.6 is a scenario with lowest GHG emissions and the most stringent climate policy, where CH_4_ emissions are expected to reduce by 40% over the century. RCP 4.5 and 6.0 represents more intermediate mitigation scenario, with low baseline and medium air pollution. RCP 8.5 represents the relatively conservative business as usual scenario with a relatively high emission scenario, with high baseline and medium to high air pollution (Van Vuuren et al., 2011). This scenario could well exemplify the upper limit as worst case scenario. Hence RCP 4.5 and RCP 8.5 were selected in studying the future climate change scenarios for the current research. Previously, Chen et al. (2016) has also measured vulnerability and adaptation gap the US cities using these two RCPs.

In order to spatially analyse the future climate impacts and vulnerabilities of Indian cities, we retrieved the RCP 4.5 and 8.5 climate scenarios from the Coordinated Regional Downscaling Experiment (CORDEX). CORDEX is a part of the World Climate Research Program (WCRP), aimed at developing an improved framework for generating regional-scale climate projections for impact assessment and adaptation studies worldwide within the IPCC AR5 timeline and beyond. Its GCMs provide reliable climate information at global scales of around 1000 x 1000 km which is relatively coarse resolution. However, for practical purposes of planning, countries require information at much finer scales than GCM. Regional Climate Models (RCMs) derive information from GCMs but at much higher resolution and for a small/ limited area of interest. CORDEX performs regional downscaling of the GCM outputs in 13 domains across the globe. India is covered by the South Asia domain and the data is provided by the Indian Institute of Tropical Meteorology (IITM) on their climate data portal (IITM 2018). The downscaled data was extracted using Data Extraction tools to access authorized datasets available on Earth System Grid Federation (ESGF) in the framework of CORDEX South Asia simulations. The downloaded data were in Net CDF data formats, with interpolated grid resolution of 0.44 degrees (WAS-44i). For details about the data, see Table A1.

Table A1: Details regarding the climate data used for scenario mapping

| **Institute** | **IITM** | | |
| --- | --- | --- | --- |
| RCM model | RegCM4-4 | | |
| Driving model | CCCma-CanESM2: CCCma Institute (Canadian Centre for Climate Modelling and Analysis, Victoria, BC, Canada) | | |
| Variables | Precipitation | PR | kg m^-2^ s^-1^(converted to mm/day) |
|  | Near Surface Air Temperature | TAS | Kelvins (K)(converted to °C) |
|  | Daily Minimum Near Surface Air Temperature | TASMIN | Kelvins (K)(converted to °C) |
|  | Daily Maximum Near Surface Air Temperature | TASMAX | Kelvins (K)(converted to °C) |

**1.2 Mapping the temperature and precipitation changes**

The mapping analysis was performed in GIS software Arc Map (version 10.2) for different time frames and spatial scales. The two RCP scenarios (4.5 and 8.5) were retrieved for three time frames: short term (2030s), mid-term (2050s) and long-term (2080s). In addition to future scenarios, the historical data ranging from year 1950 to 2005 was also extracted, processed using Pre-processing, Statistical processing and Pattern analysis (elaborated below); and analysed, (i) to understand the trends and variabilities of various climatic variables; and (ii) to estimate anomalies in temperature and precipitation under different scenarios and varying time frames.

*Pre-processing* - For extraction, primarily the datasets were cropped and masked with administrative boundaries of India based on the Survey of India Maps (Joseph et al. 2020), and then converted to relevant units

*Statistical processing* – Mean for each time series dataset was calculated to estimate mean future anomalies based on the historical variable values.

*Pattern analysis* – The outputs were analysed to study spatial distribution of change in the variables over the nation and an overall comparison for country averages was also performed for different climate scenarios with respect to historical values. This information was used to understand a general pattern of changing climate and their implications for different geographical areas of the country.


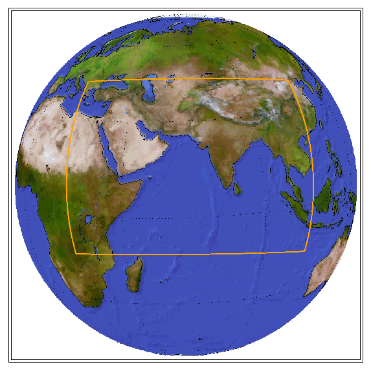

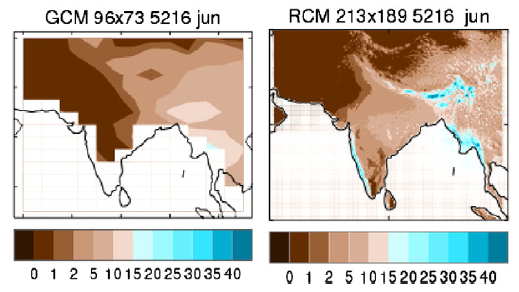


Figure A1: CORDEX South Asia domain (Region 6), and GCM vs. RCM precipitation climatology for India land mask^[[2]](#footnote-2)^

**1.3 Superimposing the geo-locations of major urban settlements**

Upon a detailed analysis of downscaling climate scenario RCP 4.5 & 8.1 (temperature and precipitation changes in each state for 2030, 2050 & 2080), major urban settlements in each state were overlaid on the to visualize the climate vulnerability of these cities. All towns and cities defined as Class-1 (with population < 100,000 by the last enumeration under Census of India 2011) were selected for this purpose. The classified Class-1 data structures are imported into Arc Map (version 10.2) and the method of super-imposition of urban settlements follows Sethi (2015). The geographic location is authenticated and updated from Google Maps 2019 (open data source) for each city. The state/regional analysis is mapped for forecast temperature (in °C) and precipitation (in mm/day) and interpreted for the variability against the historical trends (S2 Appendix). The coalesced implications at the country level are assessed and presented in Figure 2 and Figure 3 (main paper) respectively to compare future trends.

**References**

Chen, C., Doherty, M., Coffee, J., Wong, T., & Hellmann, J. (2016). Measuring the adaptation gap: A framework for evaluating climate hazards and opportunities in urban areas. *Environmental Science & Policy*, *66*, 403-419.

IITM (2018). Climate data portal. Centre for Climate Change Research. Indian Institute of Tropical Meteorology <http://cccr.tropmet.res.in/home/old_portals.jsp>

IPCC (2014) Climate Change 2014: Synthesis Report. Contribution of Working Groups I, II and III to the Fifth Assessment Report of the Intergovernmental Panel on Climate Change [Core Writing Team, R.K. Pachauri and L.A. Meyer (eds.)]. IPCC, Geneva, Switzerland, 151 pp

Mathison, C., Wiltshire, A., Dimri, A. P., Falloon, P., Jacob, D., Kumar, P., ... & Yasunari, T. (2013). Regional projections of North Indian climate for adaptation studies. *Science of the Total Environment*, 468, S4-S17.

Sethi, M. (2015). Location of greenhouse gases (GHG) emissions from thermal power plants in India along the urban-rural continuum. *Journal of Cleaner Production*, 103, 586-600.9

Joseph. K. J. V., Mozumdar A., Lhungdim H., Acharya R. (2020) Quality of care in sterilization services at the public health facilities in India: A multilevel analysis. PLoS ONE 15(11): e0241499. https://doi.org/10.1371/journal.pone.0241499

Van Vuuren, D. P., Edmonds, J., Kainuma, M., Riahi, K., Thomson, A., Hibbard, K., ... & Masui, T. (2011). The representative concentration pathways: an overview. *Climatic change*, *109*(1-2), 5.

1. Radiative forcing, expressed as Watts per square metre, is the additional energy taken up by the Earth system due to the enhanced greenhouse effect. More precisely, it can be defined as the difference in the balance of energy that enters the atmosphere and the amount that is returned to space compared to the pre-industrial situation. [↑](#footnote-ref-1)
2. Mathison et al., 2013 [↑](#footnote-ref-2)
