# Supplementary material for: How to tackle complexity in urban climate resilience? Negotiating climate science, adaptation and multi-level governance in India
Source: PLoS One. 2021 Jul 1;16(7):e0253904. doi: 10.1371/journal.pone.0253904 (PMC8248603; doi:10.1371/journal.pone.0253904)
Supplement: S2 Appendix — (DOCX) [file pone.0253904.s002.docx]

**S2 Appendix. Forecasted climate variability in temperature (in °C) and precipitation (in mm/day) against historic trends across different states/ regions of India**

**Andhra Pradesh and Telangana:**

**
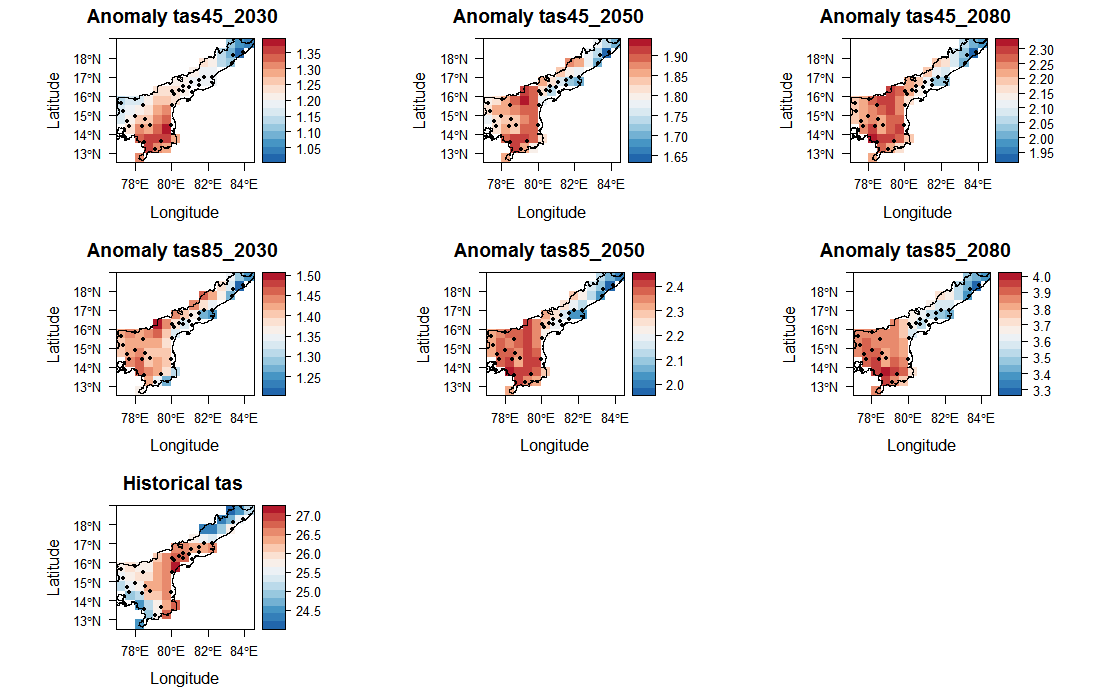
**

**Map 3.1: Andhra Pradesh – Temperature (Avg.) anomalies (°C) against historical trend**

**
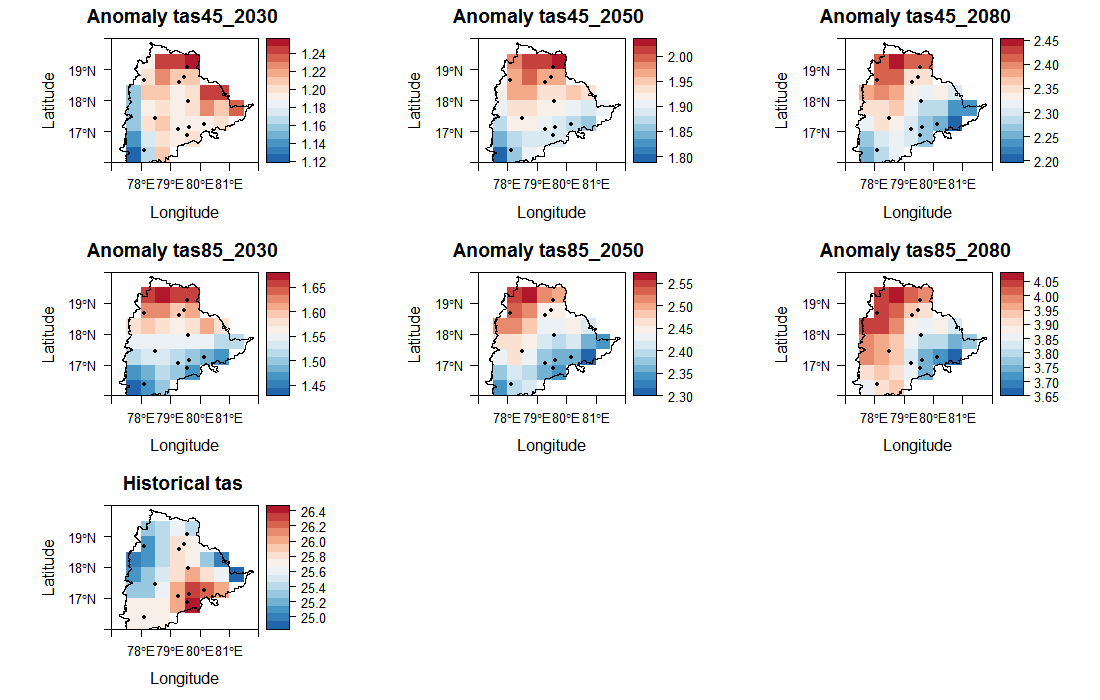
**

**Map 3.2: Telangana – Temperature (Avg.) anomalies (°C) against historical trend**

**
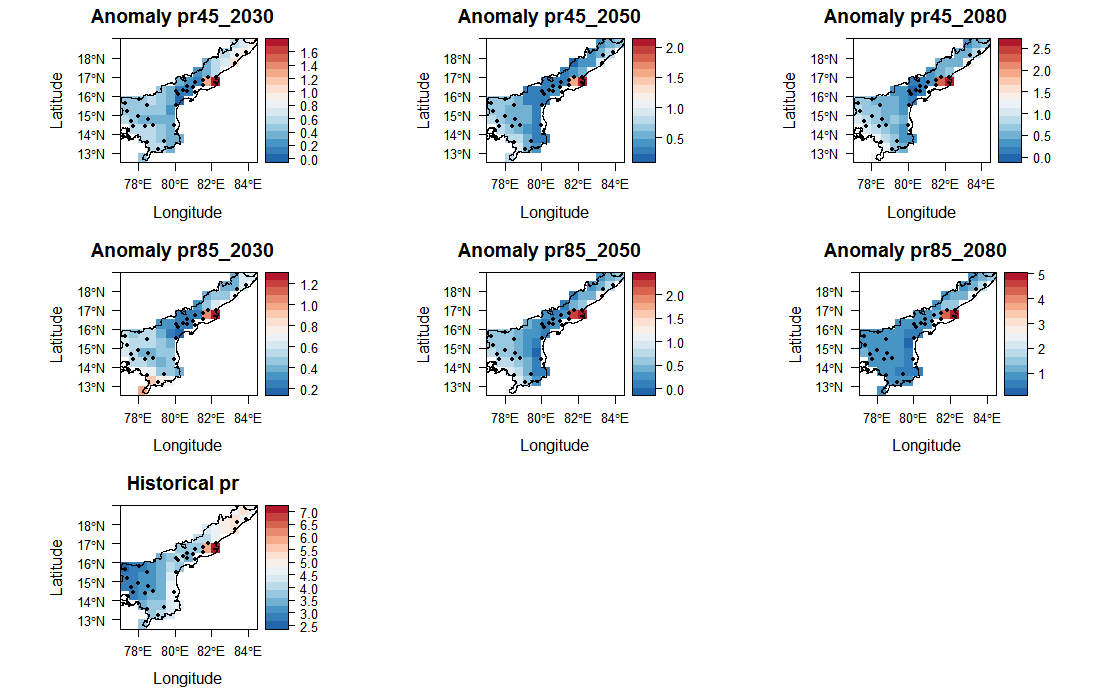
**

**Map 3.3: Andhra Pradesh – Precipitation anomalies (mm/day) against historical trend**


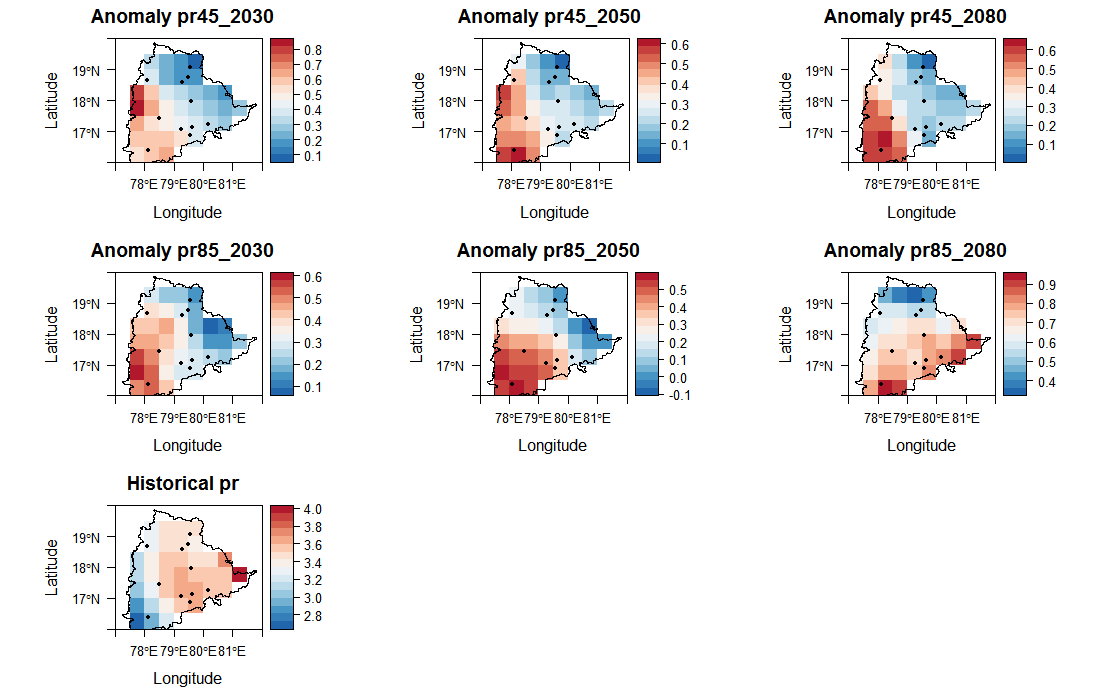


**Map 3.4: Telangana – Precipitation anomalies (mm/day) against historical trend**

| **Region/ State** | **Climate variations** | | |
| --- | --- | --- | --- |
|  | **Temperature** | **Precipitation** |  |
| **Andhra Pradesh & Telangana** | Estimates for average annual temperature increase for both RCP4.5 and RCP8.5 in the region, ranging between +1.12-1.35°C (2030s), 1.65-2.00°C (2050s) and 1.95-2.45°C (2080s) and for RCP8.5: 1.25-1.65°C (2030s), 2.00-2.55°C (2050s), 3.30-4.05°C (2080s). The Northern part of the region, in general, shows marginally greater rise in temperature in both RCP scenarios and for all time positions, than Southern part. | As per estimates, under RCP 4.5, there is a marginal increase in precipitation in the region, ranging from 0.0 to 1.6 mm/day (2030s), to 0.0 to 2.0 mm/day (2050s), to 0.0 to 2.5 mm/day (2080s). RCP8.5 scenario also shows a similar variation in average precipitation, ranging from 0.1-1.2 mm/day (2030s), to -0.1 to +2.0 mm/day (2050s), to 0.4 to +4.0 mm/day (2080s). |  |

**Assam and North Eastern States**


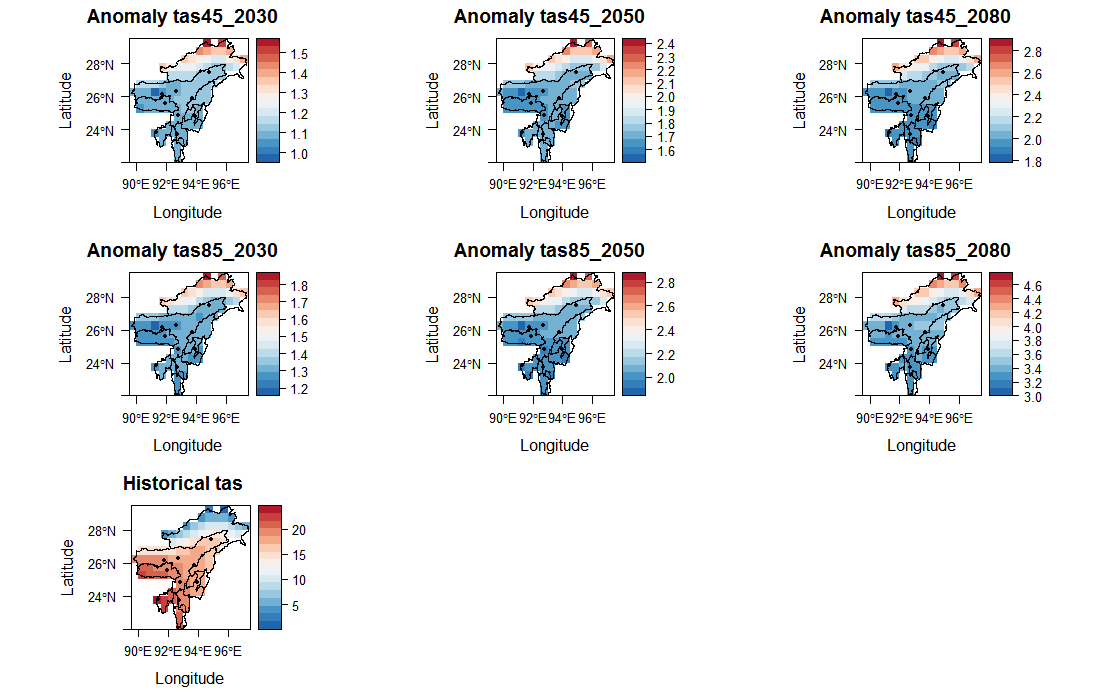


**Map 3.5: North Eastern states– Temperature (Avg.) anomalies (°C) against historical trend**


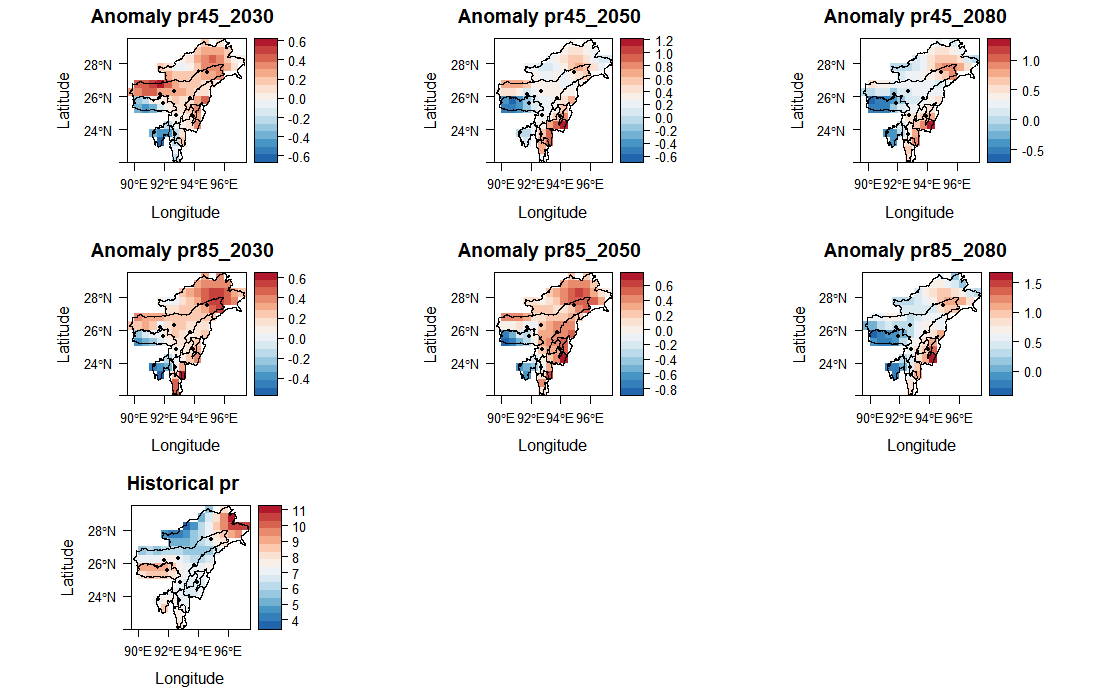


**Map 3.6: North-Eastern states – Precipitation anomalies (mm/day) against historical trend**

| **Region/ State** | **Climate variations** | |
| --- | --- | --- |
|  | **Temperature** | **Precipitation** |
| **North-East & Sikkim** | Average annual temperature increase under both RCP4.5 and RCP8.5 scenario in the region. The rise of temperature ranges from +1.00-1.50°C (2030s), 1.60-2.40°C (2050s) and 1.80-2.80°C (2080s) and for RCP8.5: 1.20-1.80°C (2030s), 1.95-2.80°C (2050s), 3.00-4.60°C (2080s). | As per estimates, under RCP 4.5, precipitation is showing variability in the region ranging from -0.6 to +0.6 mm/day (2030s), to -0.6 to +1.2 mm/day (2050s), to -0.5 to +1.5 mm/day (2080s). Similarly, RCP 8.5 scenario also shows variability in average precipitation, ranging from -0.4 to +0.6 mm/day (2030s), to -0.8 to +0.6 mm/day (2050s), to -0.5 to +1.5 mm/day (2080s). |

**Bihar**

**
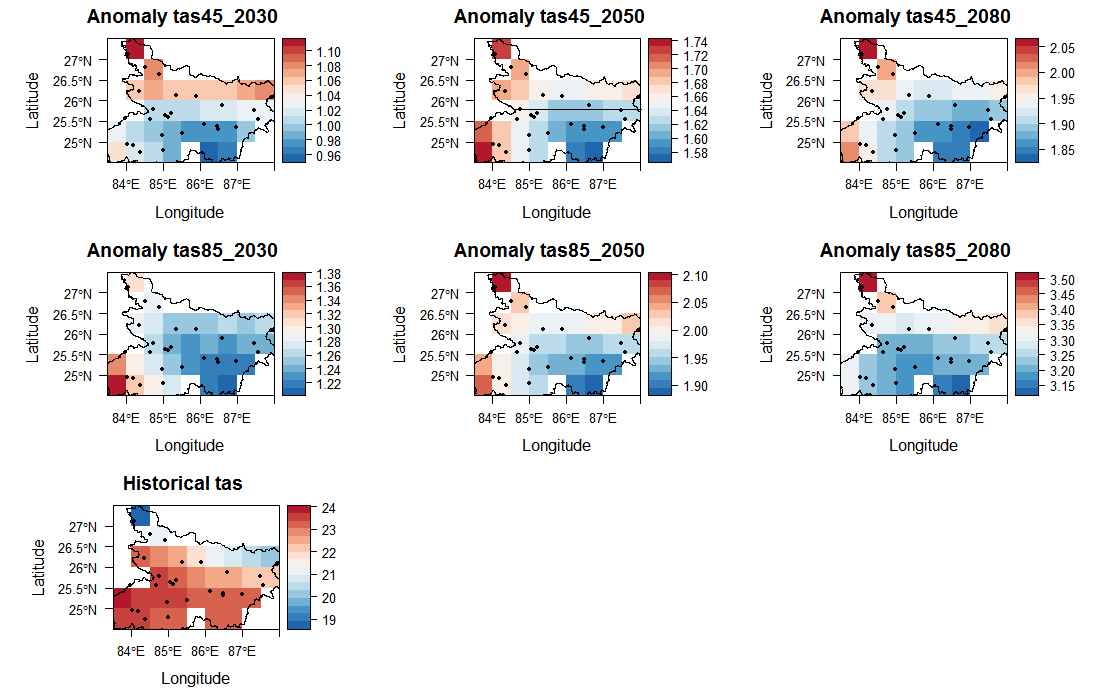
**

**Map 3.7: Bihar - Temperature (Avg.) anomalies (°C) against historical trend**


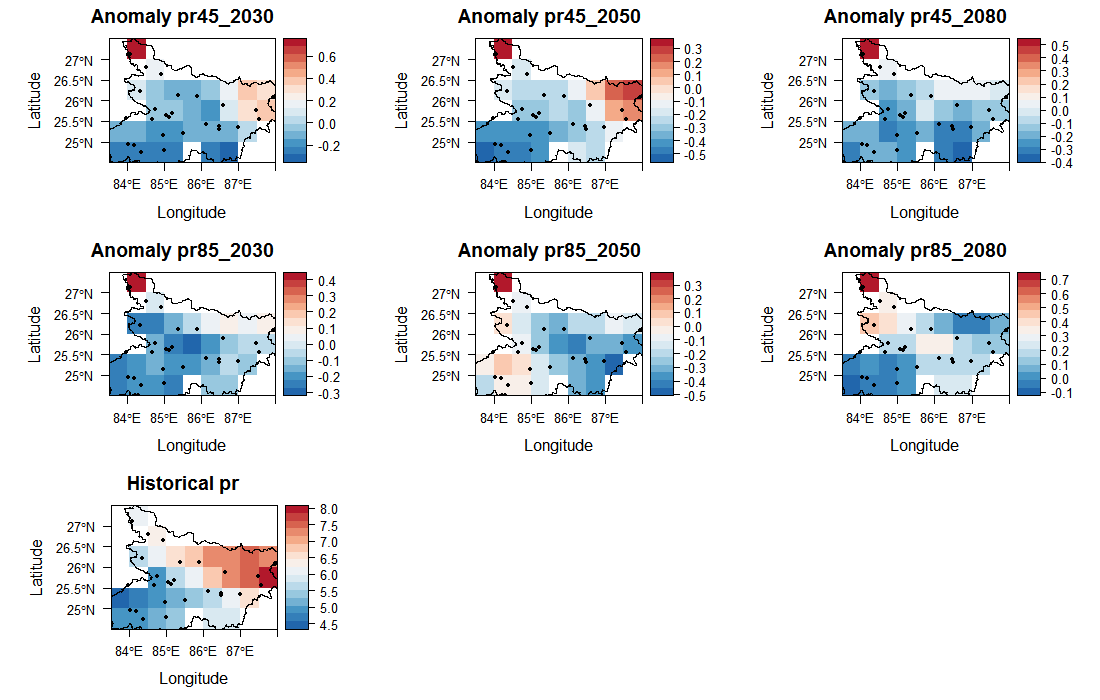


**Map 3.8: Bihar - Precipitation anomalies (mm/day) against historical trend**

| **Region/ State** | **Climate variations** | |
| --- | --- | --- |
|  | **Temperature** | **Precipitation** |
| **Bihar** | Estimates for average annual temperature increase under RCP4.5 in the state ranging between 0.96-1.10°C (2030s), 1.58-1.74°C (2050s) and 1.85-2.05°C (2080s) and for RCP8.5: 1.22-1.48°C (2030s), 1.90-2.10°C (2050s), 3.15-3.50°C (2080s). In general, the Northern part of the state shows marginally greater rise in temperature in both RCP scenarios and for all time positions, than Southern part. | As per estimates, under RCP 4.5, precipitation is showing variable trends in the state ranging from -0.1 to 0.6 mm/day (2030s), to -0.5 to +0.4 mm/day (2050s), to -0.4 to +0.5 mm/day (2080s). Similarly, RCP 8.5 scenario also shows similar variability in average precipitation, ranging from -0.3 to 0.4mm/day (2030s), to -0.5 to +0.3 mm/day (2050s), to -0.1 to +0.7 mm/day (2080s) |

**Chhattisgarh**


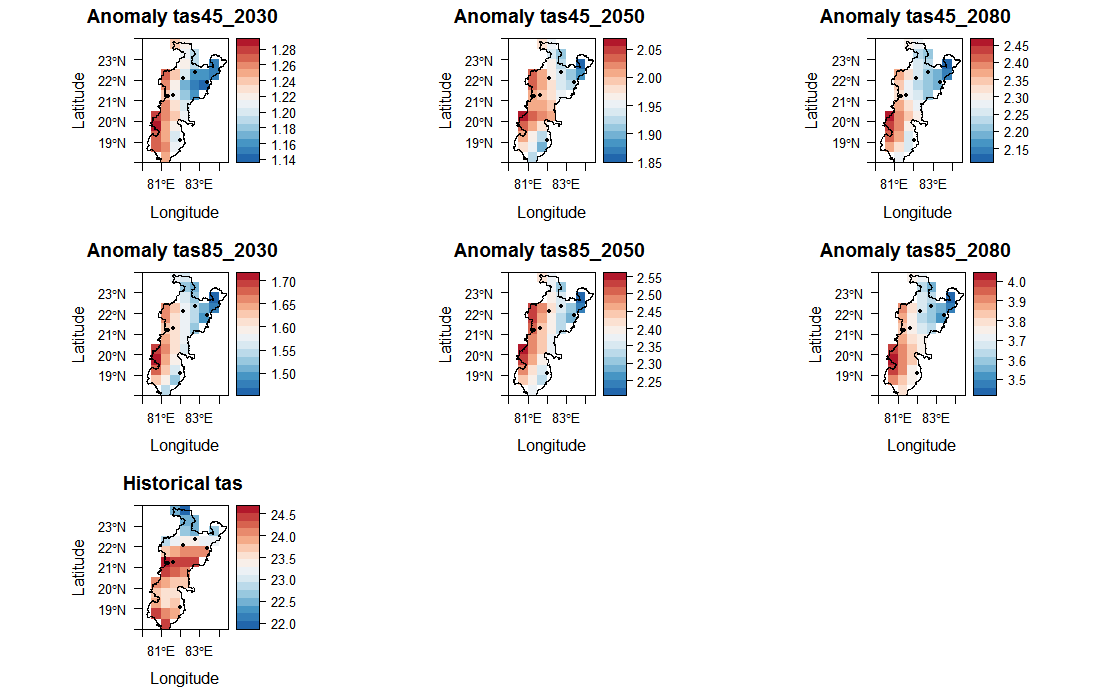


**Map 3.9: Chhattisgarh – Temperature (Avg.) anomalies (°C) against historical trend**


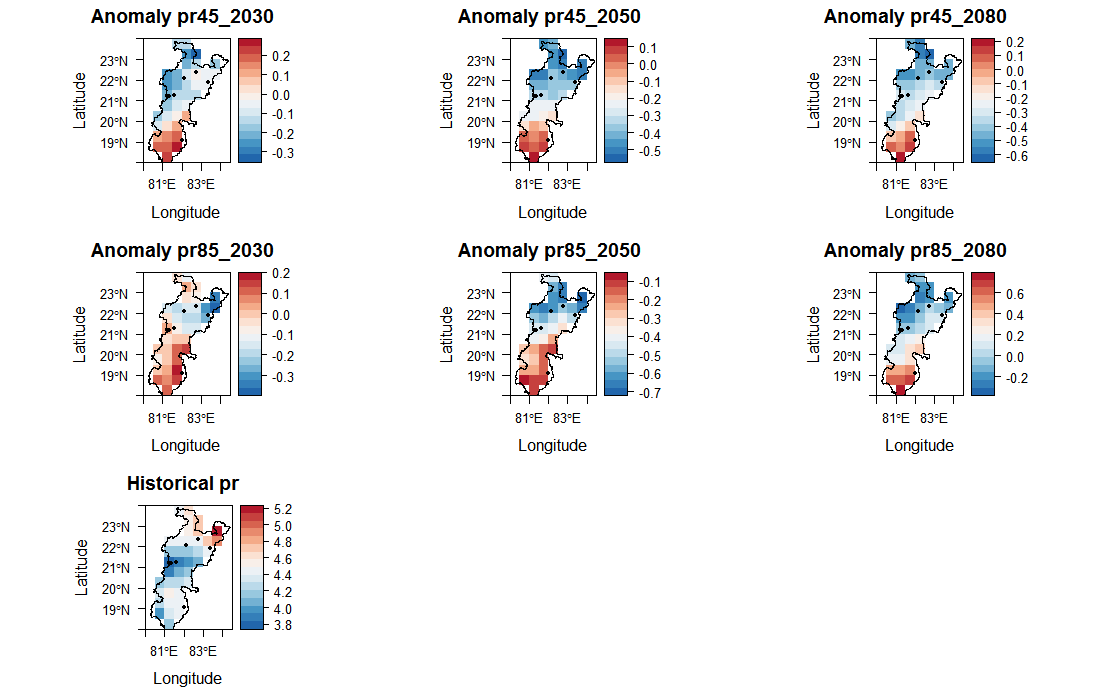


**Map 3.10: Chhattisgarh – Precipitation anomalies (mm/day) against historical trend**

| **Region/ State** | **Climate variations** | |
| --- | --- | --- |
|  | **Temperature** | **Precipitation** |
| **Chattisgarh** | Average annual temperature anomalies under RCP4.5 in the state range between 1.14-1.28°C (2030s), 1.85-2.06°C (2050s) and 2.13-2.45°C (2080s) and for RCP8.5: 1.45-1.70°C (2030s), 2.2-2.6°C (2050s), 3.4-4.05°C (2080s). In general, the Southern part of the state shows greater increase in temperature in both RCP scenarios and for all time positions, than Northern and Central part. | Under RCP 4.5, precipitation is expected to decline by 0.3mm/day (2030s) to 0.4-0.6 mm/day (2080s) in Northern Region; by 0.3mm/day (2030s) to 0.2-0.4mm/day (2080s) in Central region, while show no definite pattern in Southern region, deviating by -0.1 to 0.3 mm/day (2030s) to 0.0 to 0.2 (2080s). RCP 8.5 scenario also shows similar patterns of uncertainty in average precipitation. |

**Gujarat**


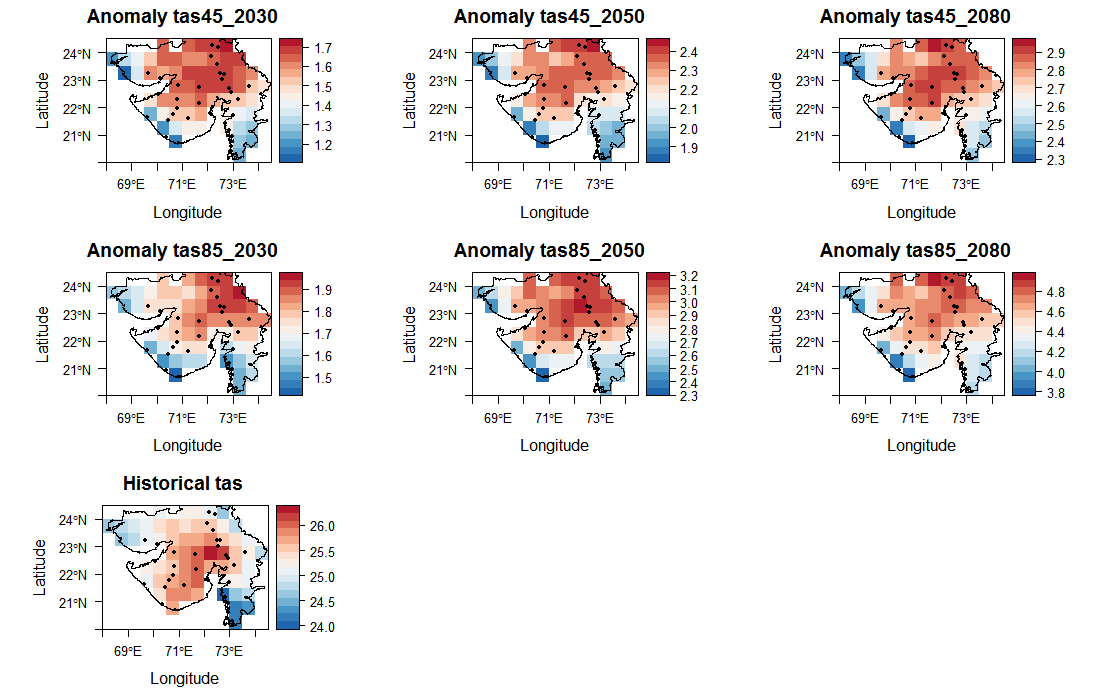


**Map 3.11: Gujarat – Temperature (Avg.) anomalies (°C) against historical trend**


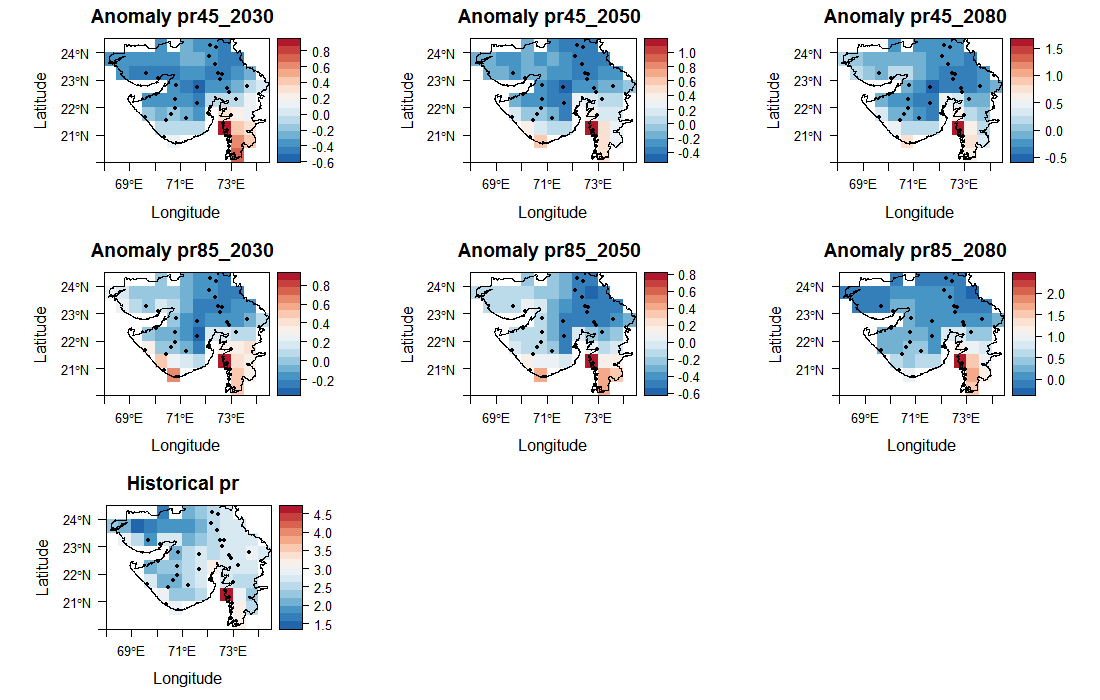


**Map 3.12: Gujarat – Precipitation anomalies (mm/day) against historical trend**

| **Region/ State** | **Climate variations** | |
| --- | --- | --- |
|  | **Temperature** | **Precipitation** |
| **Gujarat** | Projections under RCP 4.5 indicate that the temperature is set to rise by 1.3-1.7°C in Eastern Region and 1.2-1.65°C in Saurashtra (Western Region) by 2030s to up to 2.9°C by 2080s in the state. The anomalies under RCP 8.5 are even greater and rise beyond 4.8°C in 2080s | Large uncertainty in avg. precipitation from short to long term projections in both RCP4.5 and RCP 8.5 scenarios. In 2080, the anomaly in precipitation would be -0.3 to +1.5 mm/day (RCP4.5) and -0.1 to +2.5 mm/day (RCP8.5) in Eastern Region. Similarly, -0.5 to +1.0 mm/day (RCP4.5) and -0.1 to +1.0 mm/day (RCP8.5) in Saurashtra. |

**Haryana**


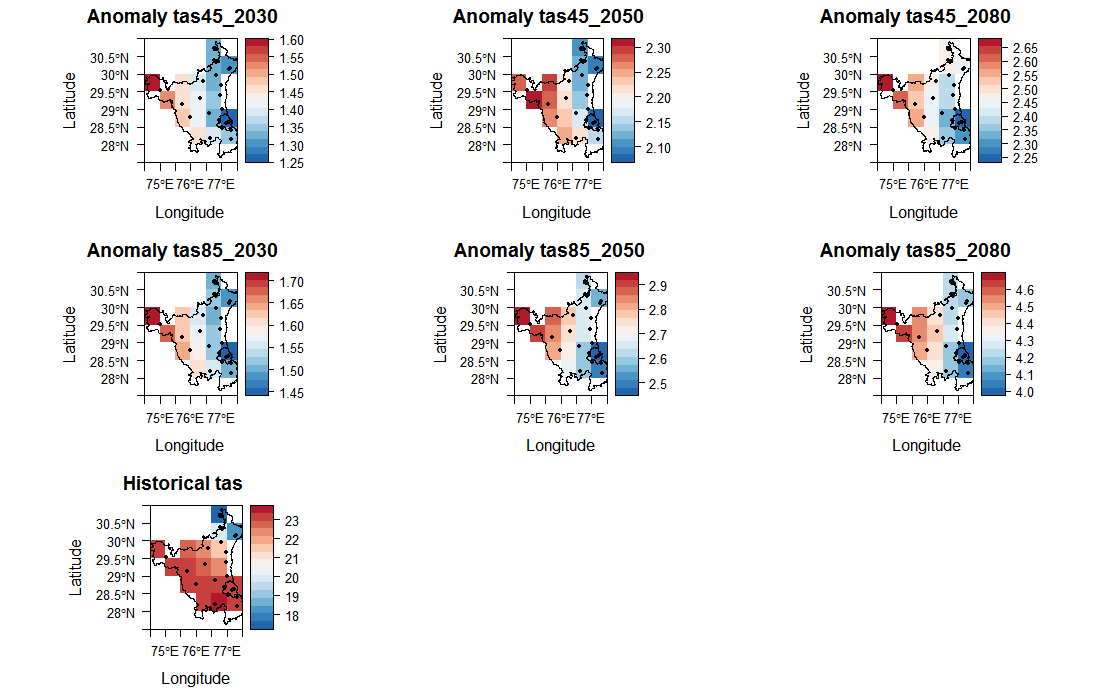


**Map 3.13: Haryana – Temperature (Avg.) anomalies (°C) against historical trend**


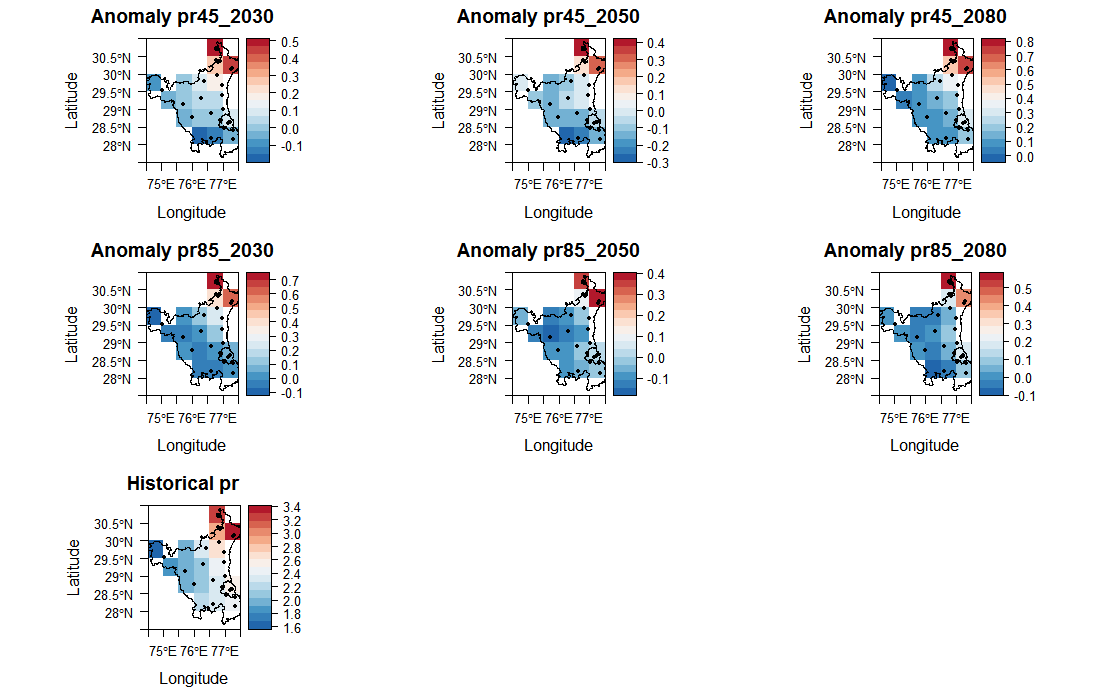


**Map 3.14: Haryana – Precipitation anomalies (mm/day) against historical trend**

| **Region/ State** | **Climate variations** | |
| --- | --- | --- |
|  | **Temperature** | **Precipitation** |
| **Haryana, Chandigarh and Delhi** | Projections under RCP 4.5 indicate that the temperature is set to rise by 1.25°C- 1.60°C by 2030s to upto 2.65°C by 2080s in the state. While southern-western region of Haryana faces highest increase in temperature in both maximum and minimum temperature as compared to northern region of the state. The anomalies under RCP 8.5 are even greater and rise beyond 4.6°C in 2080s in the entire region can be expected by 2080s. | There is Increase and decrease trend in rainfall within the state. In the western region of the state a slight decrease in overall precipitation is expected in 2030s from 0.0mm to increase of 0.1mm/day in 2080s. the northern region expected a dramatically increase in precipitation up to 0.8mm/day in 2030s to 2080s in RCP4.5 and in RCP8.5 the increase is about 0.6mm/day which is slightly lower than the RCP4.5 scenario. In both the RCPs, precipitation increases at highest rate in north- east region as compared to south-west region. |

**Himachal Pradesh**


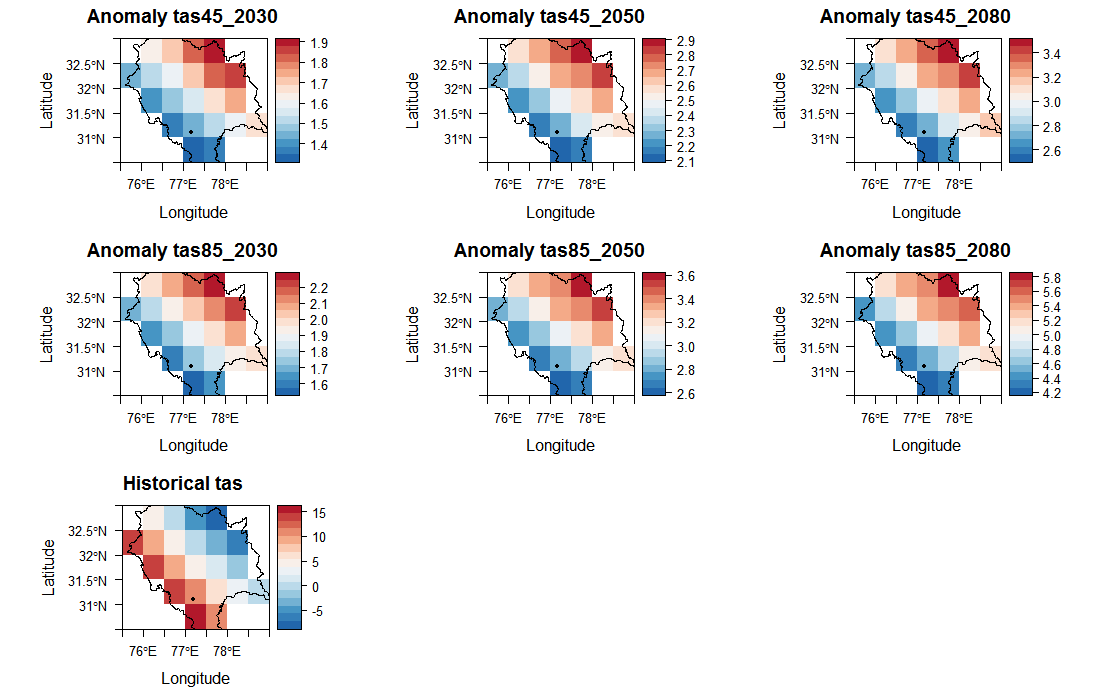


**Map 3.15: Himachal Pradesh –Temperature (Avg.) anomalies (°C) against historical trend**


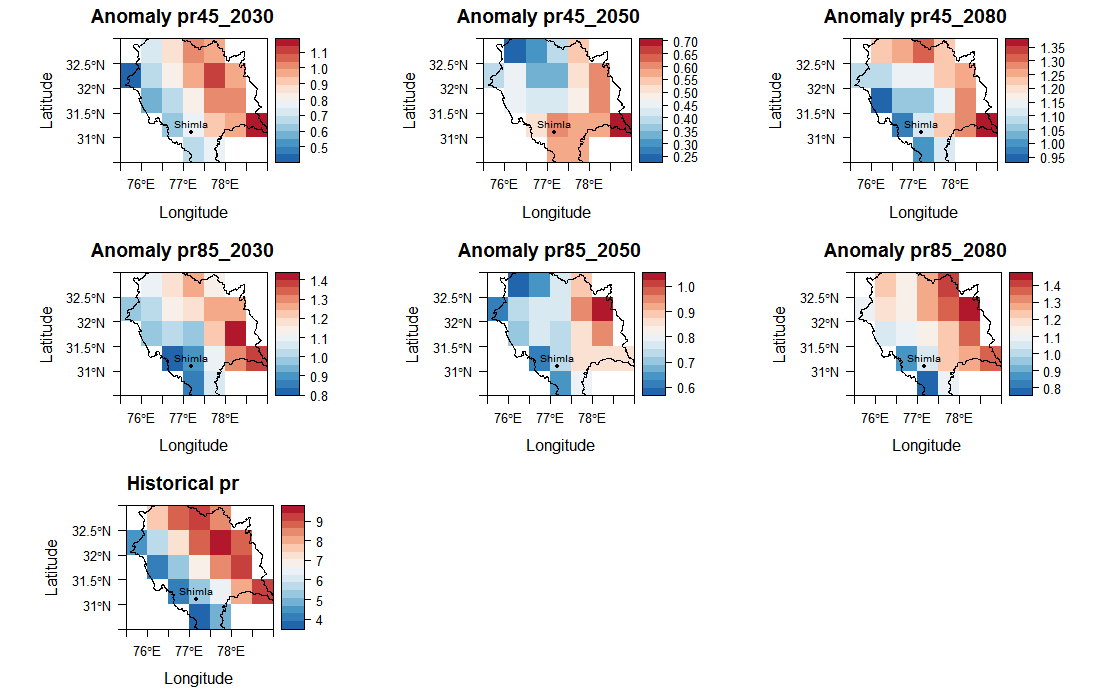


**Map 3.16: Himachal Pradesh – Precipitation anomalies (mm/day) against historical trend**

| **Region/ State** | **Climate variations** | |
| --- | --- | --- |
|  | **Temperature** | **Precipitation** |
| **Himachal Pradesh** | Projections under RCP 4.5 indicate that the temperature is expected to rise by up to 1.4°C by 2030s to up to 2.6°C in the Shivalik region; from 1.4°C to 1.6°C by 2030s to up to 3.0°C by 2080s in the middle Himalayan regions; whereas in the higher Himalayan region the rise in temperature is expected in the range of 1.7°C to 1.9°C in 2030s to up to 3.0°C to 3.4°C. The anomalies under RCP 8.5 are even greater and rise beyond 5°C in higher Himalayas can be expected by 2080s, which is an ecologically sensitive area. | Under RCP 4.5 scenario, precipitation is set to increase in the entire state from 0.5mm/day to 1.1mm/day in 2030s to up to 0.95mm/day to 1.35mm/day by 2080s. Increase in precipitation is particularly more towards higher Himalayas. |

**Jammu and Kashmir**


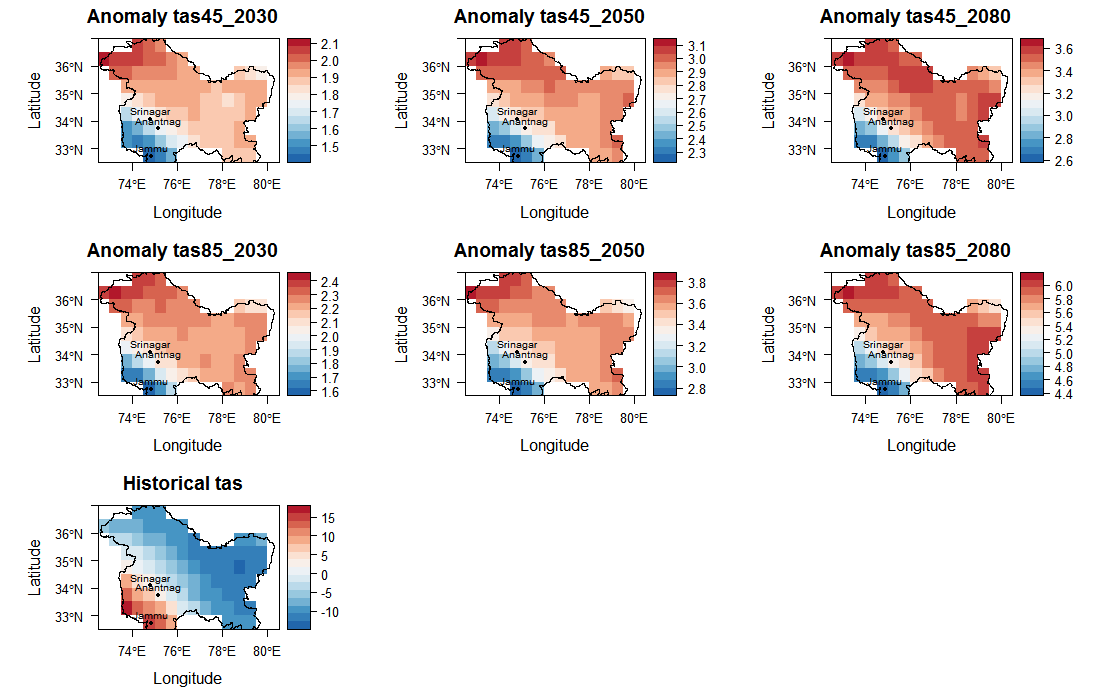


**Map 3.17: Jammu & Kashmir – Temperature (Avg.) anomalies (°C) against historical trend**


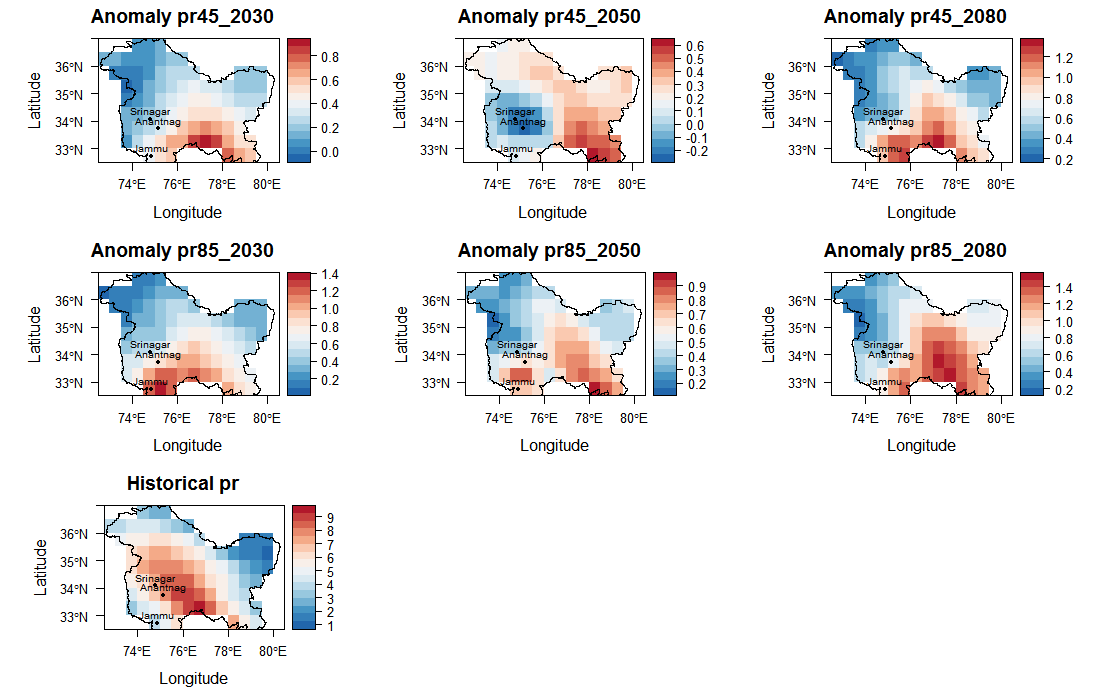


**Map 3.18: Jammu & Kashmir – Precipitation anomalies (mm/day) against historical trend**

| **Region/ State** | **Climate variations** | | |
| --- | --- | --- | --- |
|  | **Temperature** | **Precipitation** |  |
| **Jammu and Kashmir** | Projections under RCP 4.5 indicate that the temperature is set to rise by 1.9°C by 2030s to up to 3.4°C by 2080s in the trans Himalayan region, by 1.6°C by 2030s to up to 5.0°C by 2080s in the Kashmir region and by 1.5°C by 2030s to up to 4.6°C by 2080s in the Jammu region. Temperature increase– both max. & min. is highest in the trans-Himalayan Ladakh region. | Projection under RCP 4.5 indicate that the average precipitation is expected to rise in the range of 0 mm/day to 0.8mm/day in 2030s to 0.2mm/day to 1.2mm/day by 2080s in the entire state. |  |

**Jharkhand**


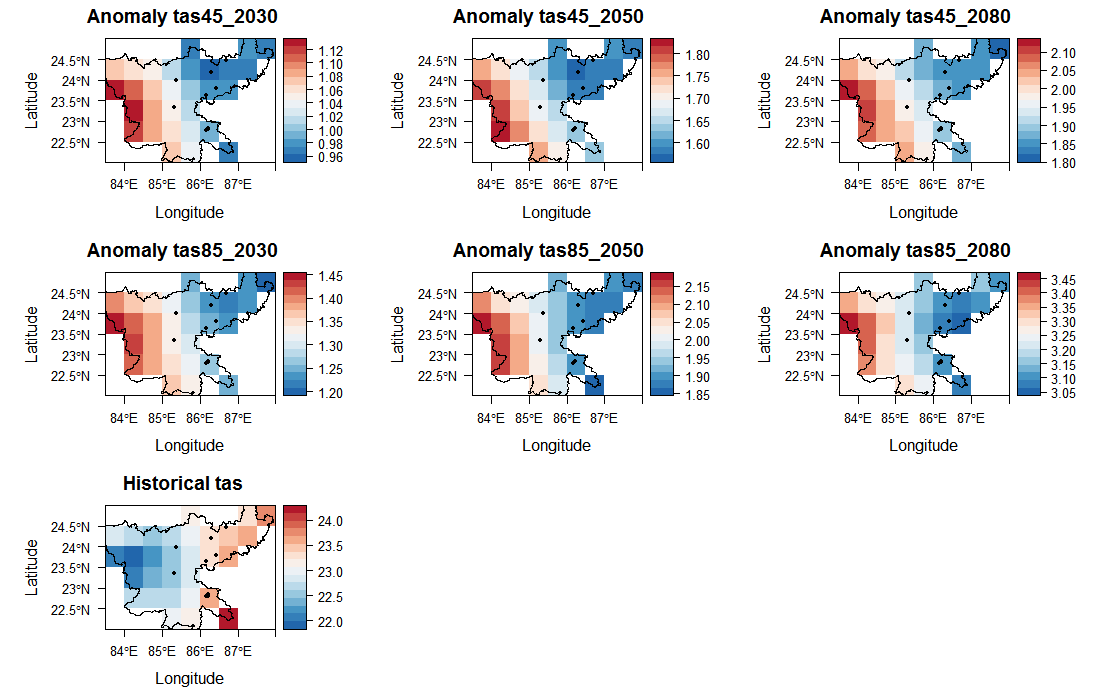


**Map 3.19: Jharkhand – Temperature (Avg.) anomalies (°C) against historical trend**


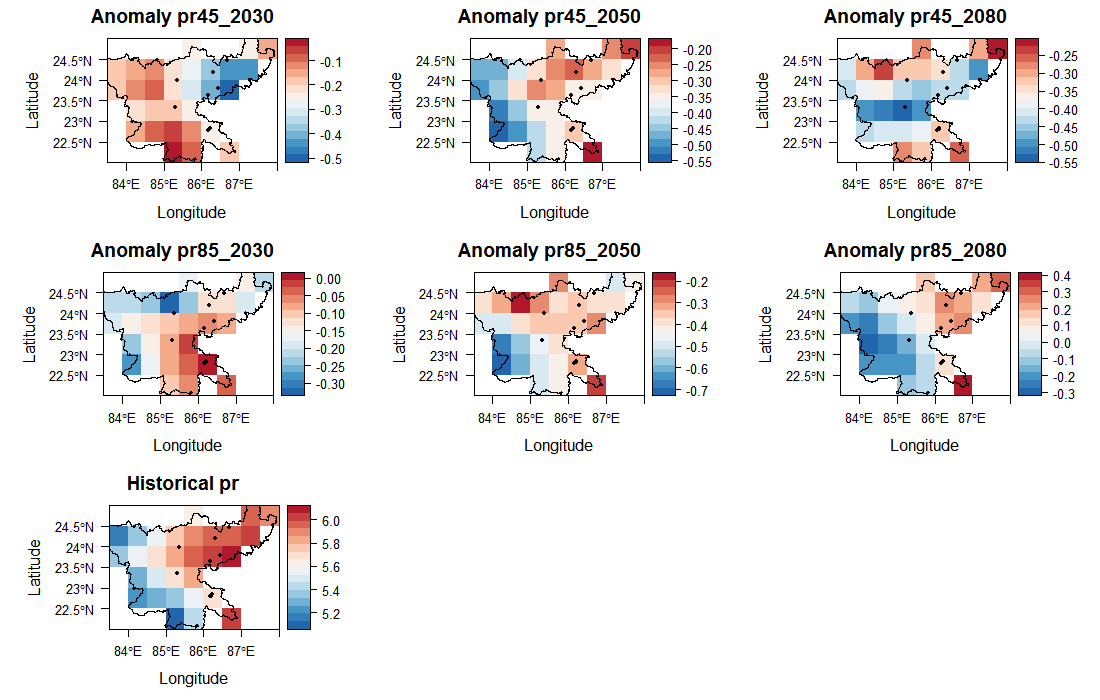


**Map 3.20: Jharkhand – Precipitation anomalies (mm/day) against historical trend**

| **Region/ State** | **Climate variations** | |
| --- | --- | --- |
|  | **Temperature** | **Precipitation** |
| **Jharkhand** | Average annual temperature increase under RCP4.5 in the state ranging between 0.96-1.12°C (2030s), 1.60-1.80°C (2050s) and 1.80-2.10°C (2080s) and for RCP8.5: 1.20-1.45°C (2030s), 1.85-2.15°C (2050s), 3.05-3.45°C (2080s). In general, the Southern part of the state shows marginally greater rise in temperature in both RCP scenarios and for all time positions, than Northern part. | Under RCP 4.5, precipitation is showing variable trends in the state ranging from -0.5 to 0.0 mm/day (2030s), to -0.55 to -0.20 mm/day (2050s), to –0.55 to -0.20 mm/day (2080s). Similarly, RCP 8.5 scenario also shows similar variability in average precipitation, ranging from -0.3 to 0.0 mm/day (2030s), to -0.7 to -0.2 mm/day (2050s), to -0.3 to +0.4 mm/day (2080s) |

**Karnataka**


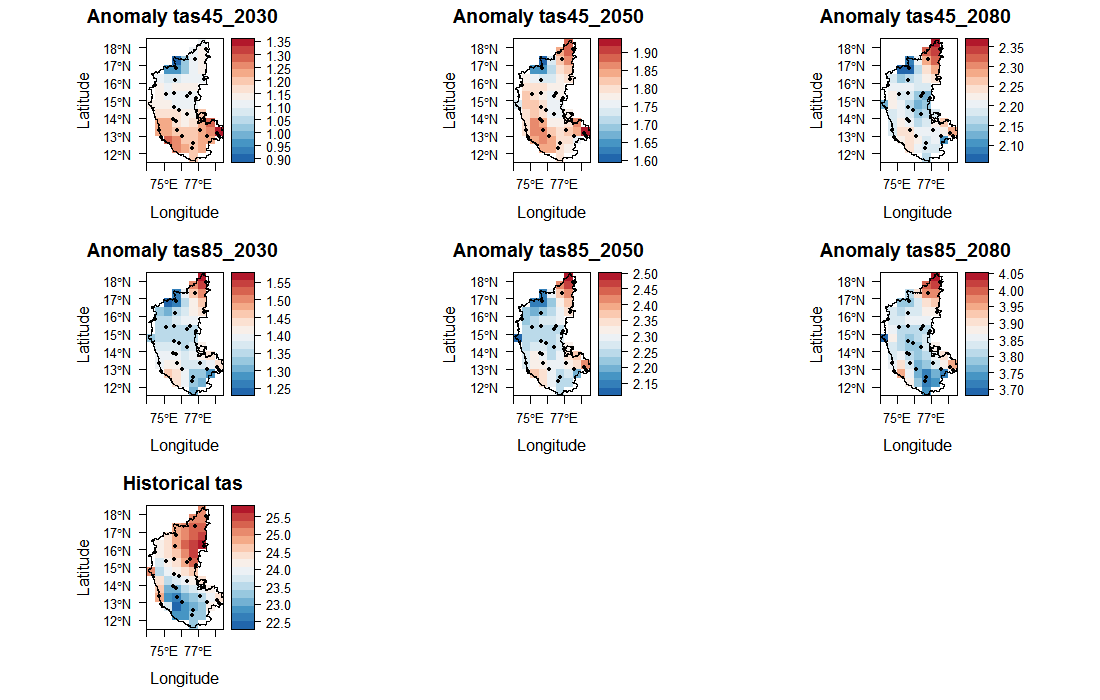


**Map 3.21: Karnataka – Temperature (Avg.) anomalies (°C) against historical trend**


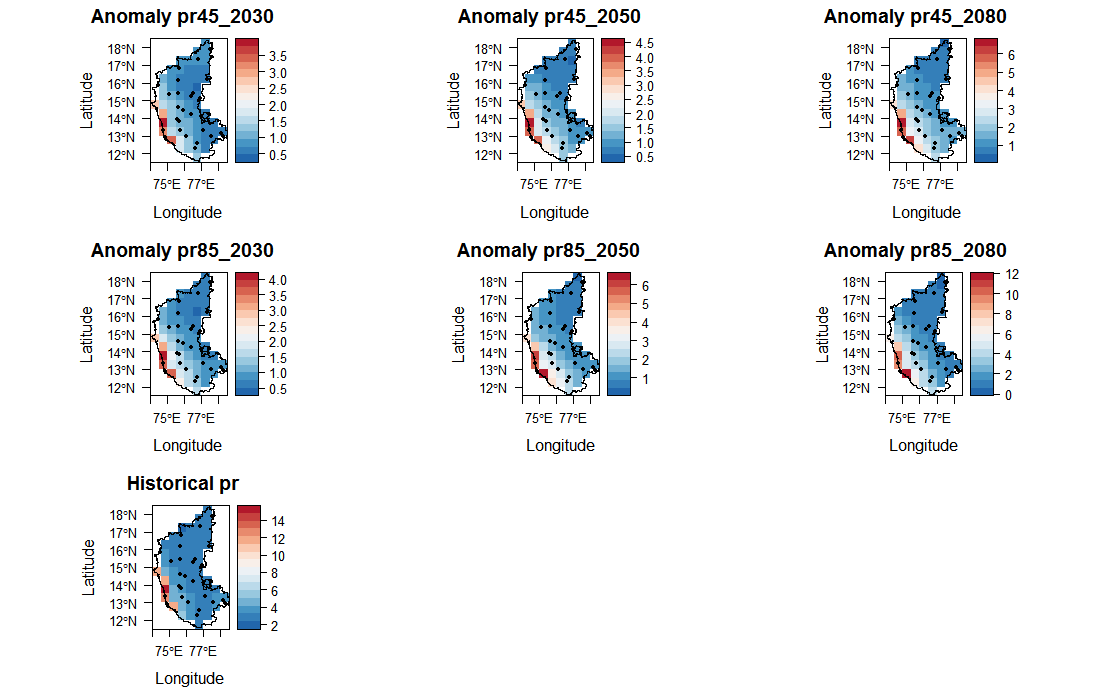


**Map 3.22: Karnataka – Precipitation anomalies (mm/day) against historical trend**

| **Region/ State** | **Climate variations** | |
| --- | --- | --- |
|  | **Temperature** | **Precipitation** |
| **Karnataka** | Average annual temperature increase under both RCP4.5 and RCP8.5 scenario in the region. The rise of temperature ranges from +0.90-1.35°C (2030s), +1.60-1.9°C (2050s) and +2.0-2.35°C (2080s) and for RCP8.5: +1.25-1.55°C (2030s), +2.15- 2.50°C (2050s), +3.70- 4.05°C (2080s). | As per estimates, under RCP 4.5, precipitation is showing an increase in the region ranging from 0.0-3.5 mm/day (2030s), to 0.0-4.5 mm/day (2050s), to 1.0-6.0 mm/day (2080s). Similarly, RCP 8.5 scenario also shows a significant increase in average precipitation, ranging from 0.5-4.0 mm/day (2030s), to 1.0-6.0 mm/day (2050s), to 1.0-12.0 mm/day (2080s). |

**Kerala**


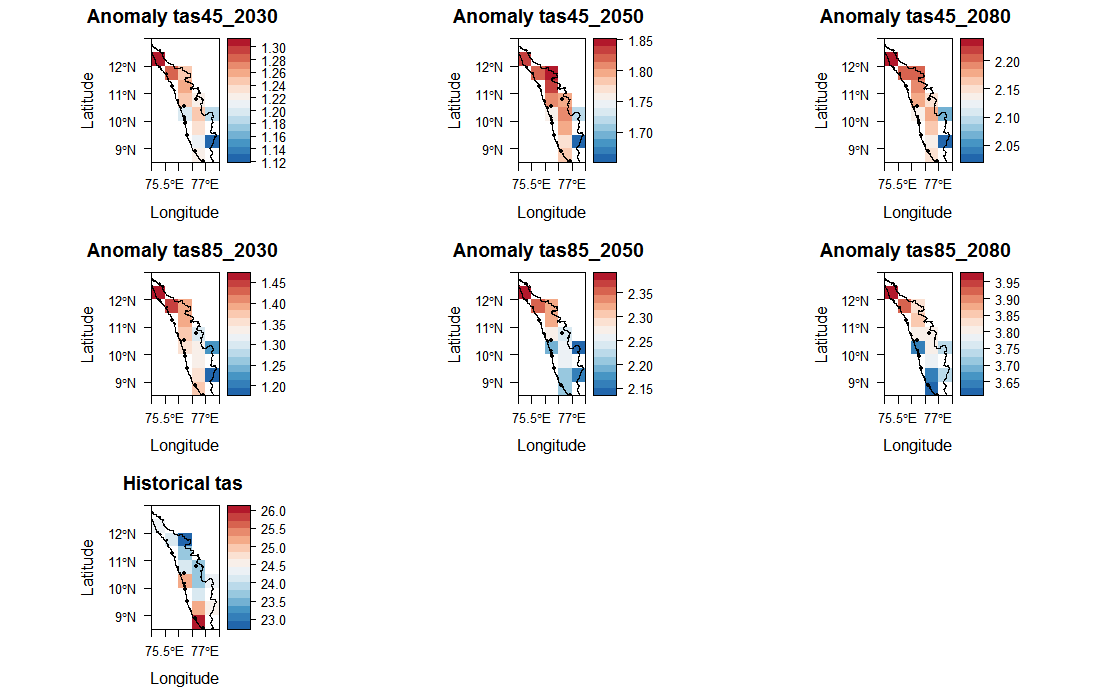


**Map 3.23: Kerala– Temperature (Avg.) anomalies (°C) against historical trend**


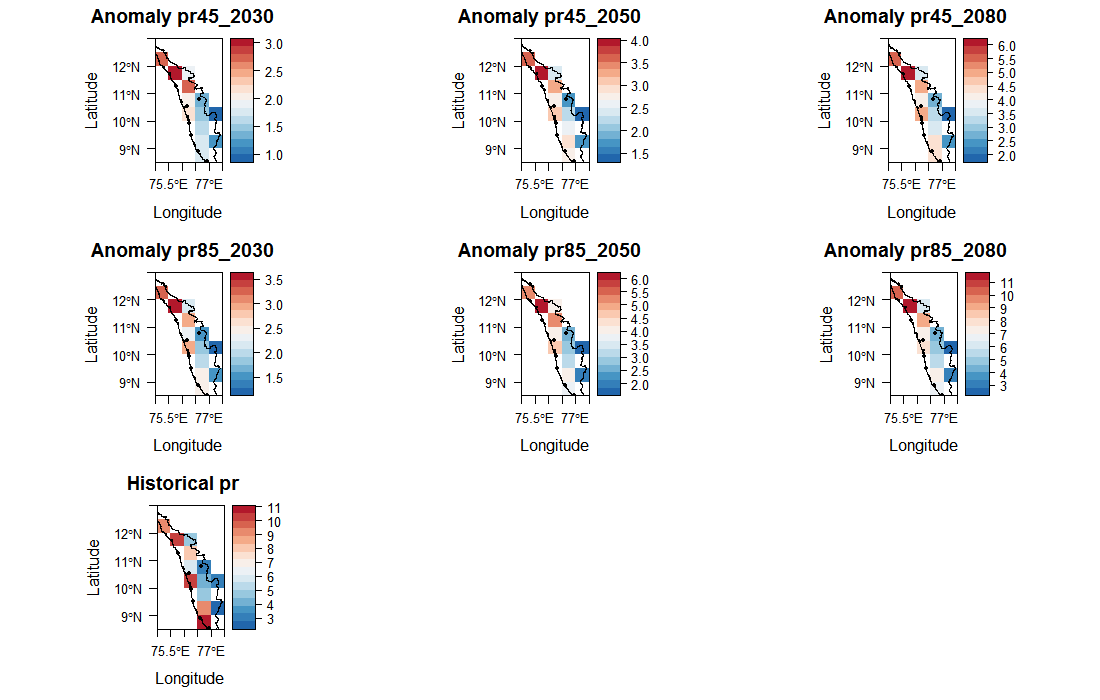


**Map 3.24: Kerala– Precipitation anomalies (mm/day) against historical trend**

| **Region/ State** | **Climate variations** | |
| --- | --- | --- |
|  | **Temperature** | **Precipitation** |
| **Kerala** | Estimates for average annual temperature increase under both RCP4.5 and RCP8.5 scenario in the state. The rise of temperature ranges from +1.12-1.30°C (2030s), 1.65-1.85°C (2050s) and 2.0-2.25°C (2080s) and for RCP8.5: 1.20-1.45°C (2030s), 2.15-2.40°C (2050s), 3.60-3.95°C (2080s). Further, projections suggest that average annual precipitation anomalies are highest in northern region of Kerala in both the scenarios. | As per estimates, under RCP 4.5, precipitation is showing an increase in the state ranging from 1.0-3.0 mm/day (2030s), to 1.5 to 4.0 mm/day (2050s), to 2.0 to 6.0 mm/day (2080s). Similarly, RCP 8.5 scenario also shows a significant increase in average precipitation, ranging from 1.0-3.5 mm/day (2030s), to 2.0-6.0 mm/day (2050s), to 3.0-11.0 mm/day (2080s) |

**Madhya Pradesh**


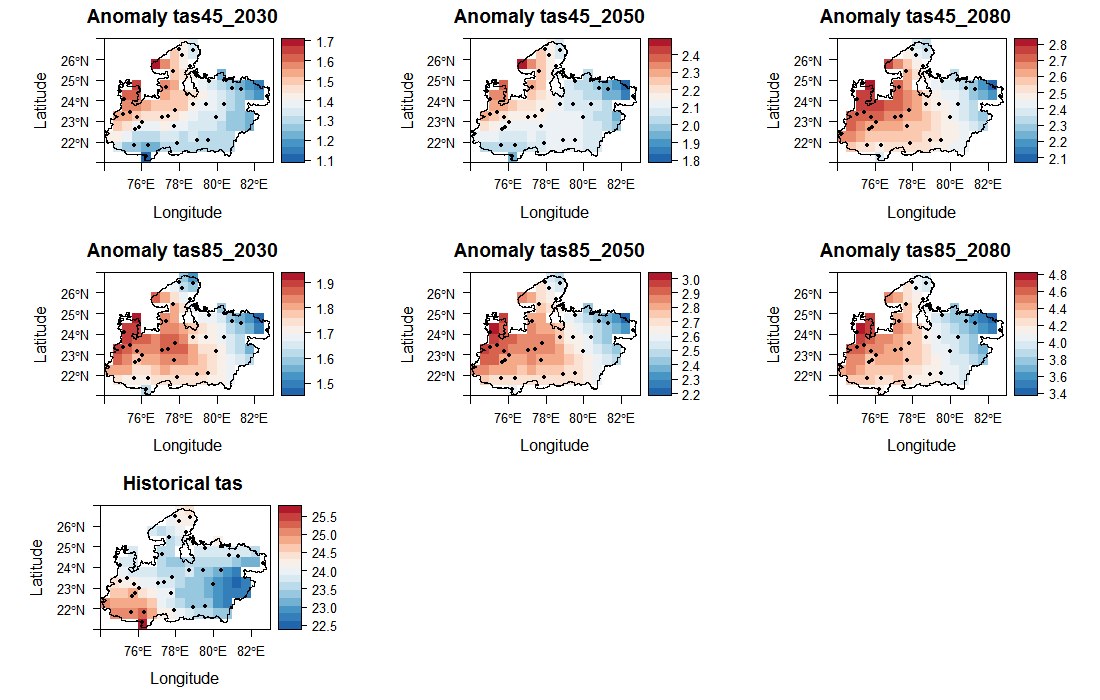


**Map 3.25: Madhya Pradesh – Temperature anomalies (°C) against historical trend**


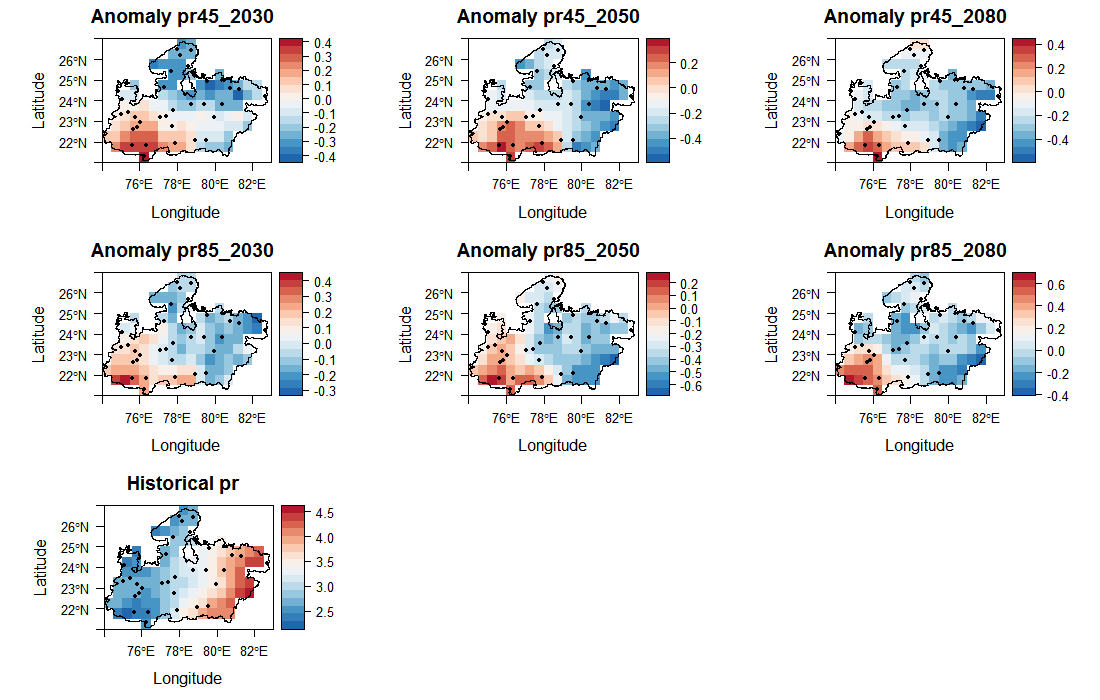


**Map 3.26: Madhya Pradesh – Precipitation anomalies (mm/day) against historical trend**

| **Region/ State** | **Climate variations** | |
| --- | --- | --- |
|  | **Temperature** | **Precipitation** |
| **Madhya Pradesh (MP)** | Average annual temperature anomalies are highest in West MP in both the RCP scenarios: RCP4.5 1.2-1.7°C (2030s), 1.9-2.4°C (2050s) and 2.4-2.8°C (2080sand RCP8.5: 1.55-1.90°C (2030s), 2.5-3.0°C (2050s), 3.9-4.8°C (2080s), although temperature anomaly from 2030-80 would remain in the range of 1.1-2.5°C in RCP4.5 and 1.5-4.1°C in RCP 8.5. This region is adjacent to eastern state of Rajasthan. Due to rise in temperature, heat waves would become intense, especially for western MP (Malwa region) and northern MP (Gird & Bundelkhand region). | Precipitation is expected to rise by 0.2-0.7 mm/day (2030s) to 0.2-0.6 mm/day (2080s) in RCP4.5 in West MP sector. Conversely, it shows uncertainty in average precipitation with anomaly of -0.1 to 0.5 (2030s) to -0.2 to 0.7 (2080s) for East MP. |

**Maharashtra**


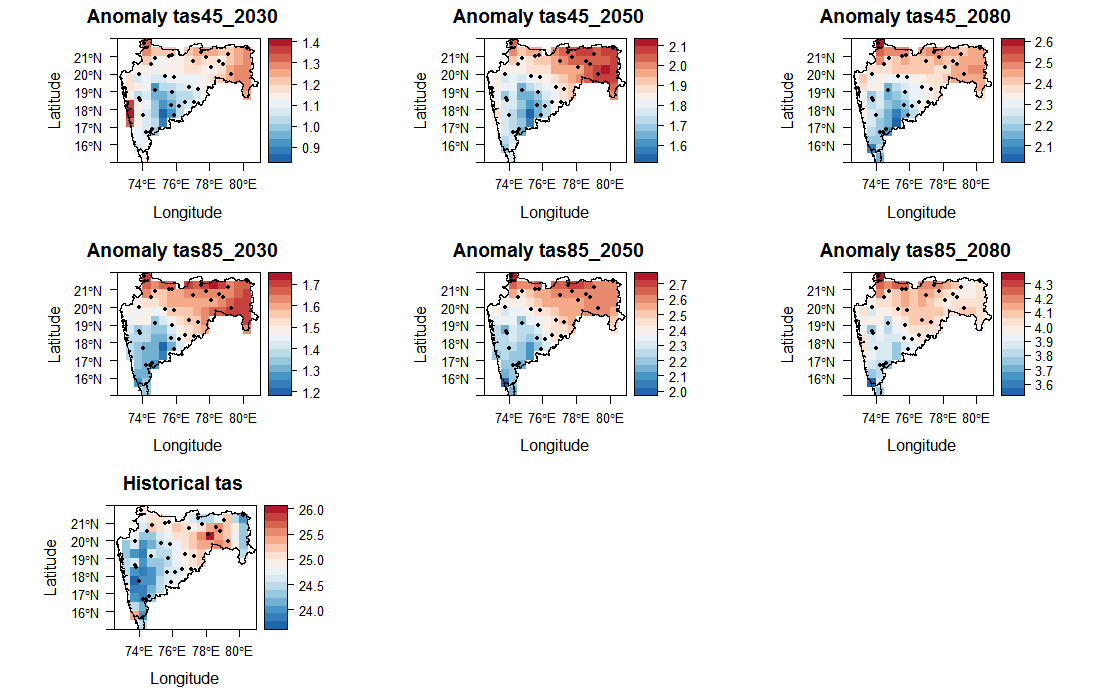


**Map 3.27: Maharashtra and Goa – Temperature (Avg.) anomalies (°C) against historical trend**


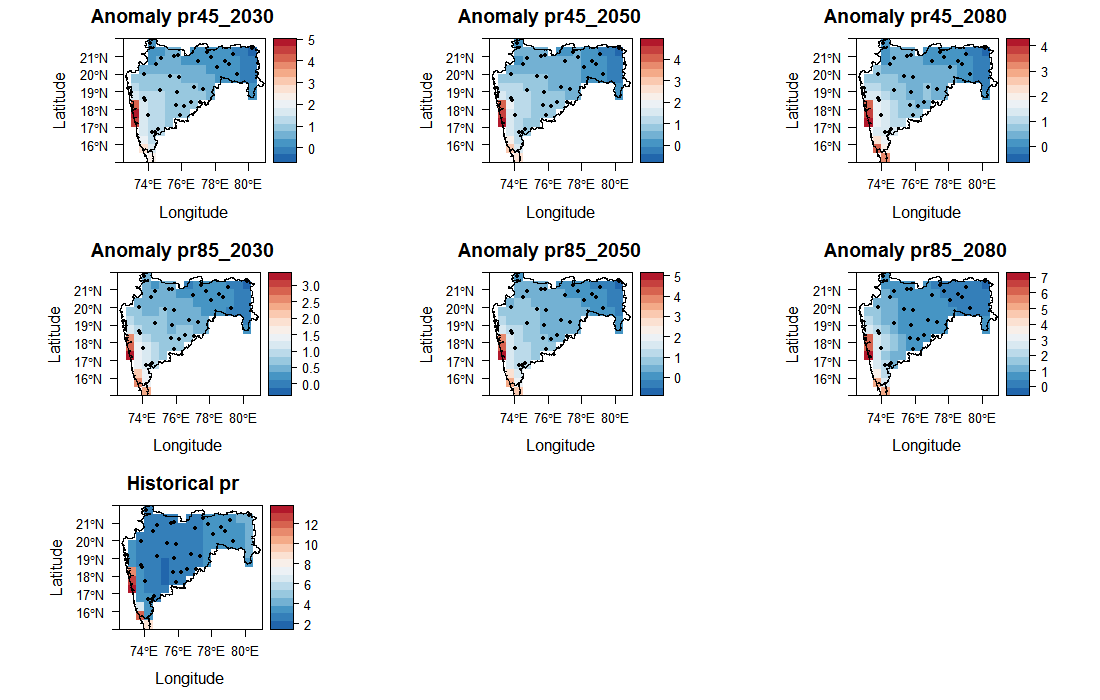


**Map 3.28: Maharashtra and Goa– Precipitation anomalies (mm/day) against historical trend**

| **Region/ State** | **Climate variations** | |
| --- | --- | --- |
|  | **Temperature** | **Precipitation** |
| **Maharashtra & Goa** | Average annual temperature increase under both RCP4.5 and RCP8.5 scenario in the region. The rise of temperature ranges from +0.90-1.4°C (2030s), +1.60-2.1°C (2050s) and +2.1-2.6°C (2080s) and for RCP8.5: +1.2-1.7°C (2030s), +2.0- 2.7°C (2050s), +3.6- 4.3°C (2080s). | As per estimates, under RCP 4.5, precipitation is showing an increase in the region ranging from 0.0-5.0 mm/day (2030s), to 0.0-5.0 mm/day (2050s), to 0.0-5.0 mm/day (2080s). Similarly, RCP 8.5 scenario also shows a significant increase in average precipitation, ranging from 0.0-4.0 mm/day (2030s), to 0.0-5.0 mm/day (2050s), to 0.0-7.0 mm/day (2080s). |

**Odisha (Orissa)**


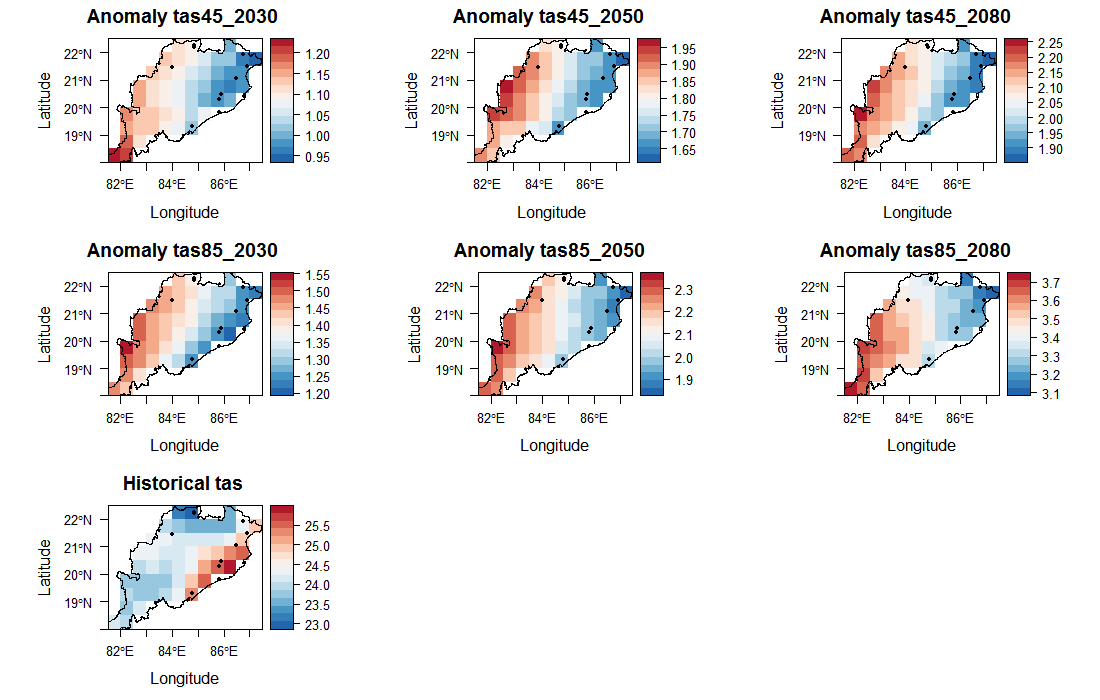


**Map 3.29: Odisha– Temperature (Avg.) anomalies (°C) against historical trend**


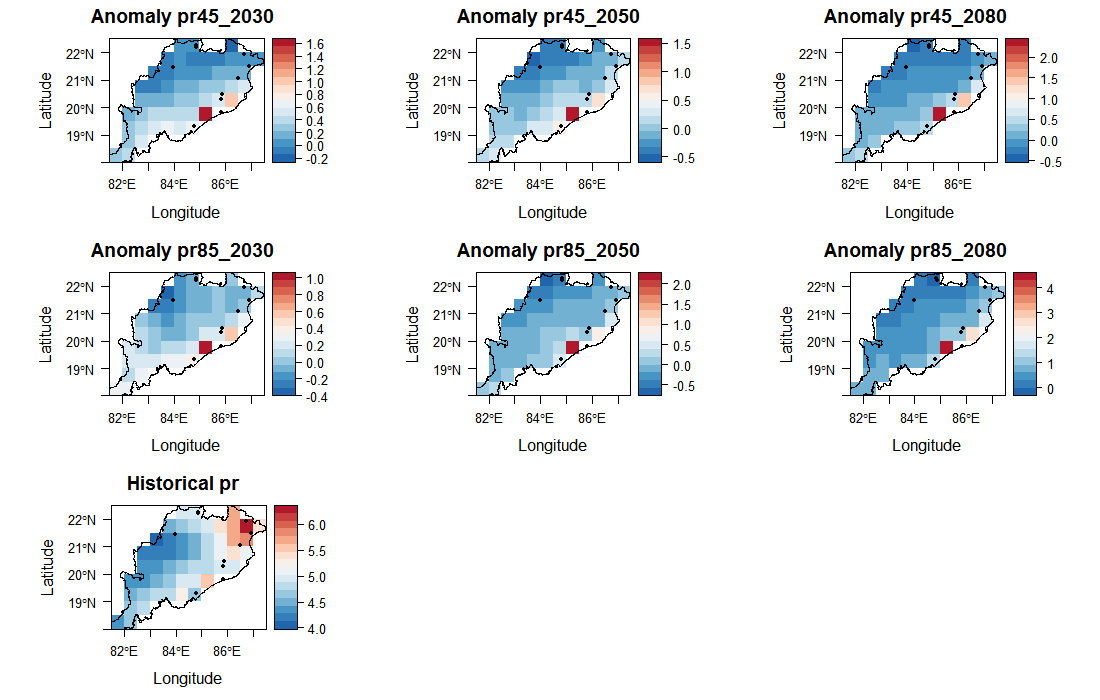


**Map 3.30: Odisha– Precipitation anomalies (mm/day) against historical trend**

| **Region/ State** | **Climate variations** | |
| --- | --- | --- |
|  | **Temperature** | **Precipitation** |
| **Odisha** | Average annual temperature increase under both RCP4.5 and RCP8.5 scenario in the region. The rise of temperature ranges from 0.95-1.20°C (2030s), 1.65-1.95°C (2050s) and 1.85-2.25°C (2080s) and for RCP8.5: 1.20-1.55°C (2030s), 1.90-2.35°C (2050s), 3.10-3.70°C (2080s). | RCP 4.5 estimates suggest variability in precipitation in the state, ranging from -0.2 to +1.6 mm/day (2030s), to -0.5 to 1.5 mm/day (2050s), to -0.5 to +2.0 mm/day (2080s). Similarly, RCP 8.5 scenario also shows variability in average precipitation, ranging from -0.4 to +1.0 mm/day (2030s), to -0.5 to +2.0 mm/day (2050s), to 0.0 to +4.0 mm/day (2080s). |

**Punjab**


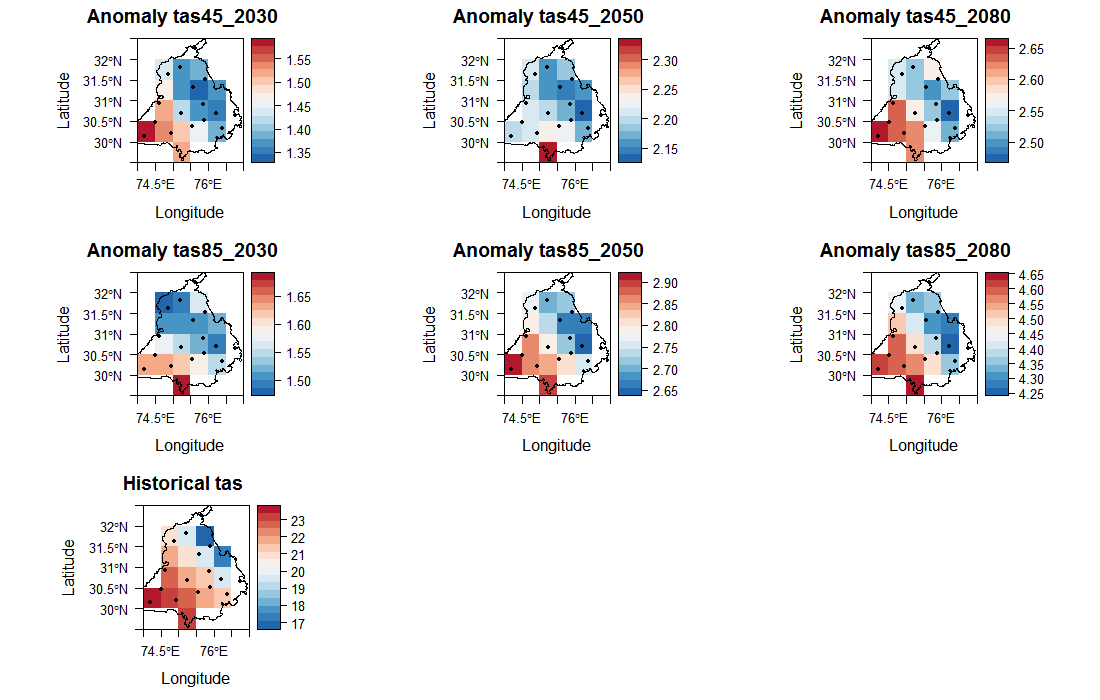


**Map 3.31: Punjab– Temperature (Avg.) anomalies (°C) against historical trend**


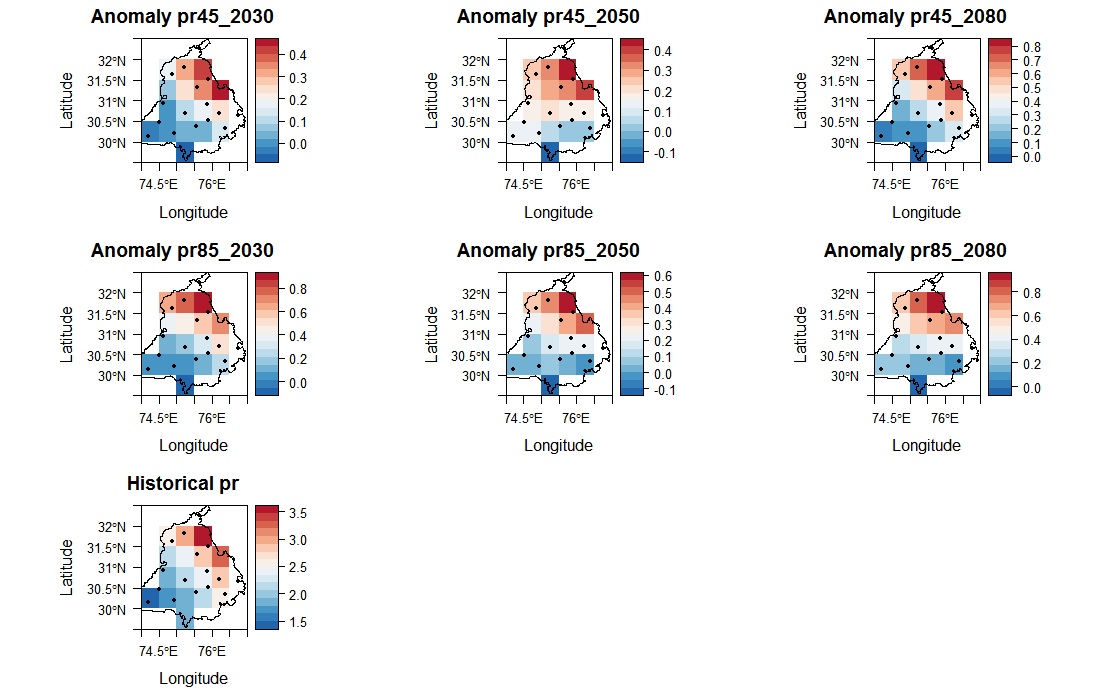


**Map 3.32: Punjab– Precipitation anomalies (mm/day) against historical trend**

| **Region/ State** | **Climate variations** | | |
| --- | --- | --- | --- |
|  | **Temperature** | **Precipitation** |  |
| **Punjab** | Projections under RCP 4.5 indicate that the temperature is set to rise by 1.35°C- 1.55°C by 2030s to up to 2.65°C by 2080s in the state. While Southern region of Punjab faces highest increase in temperature in both maximum and minimum temperature as compared to northern region of the state. The anomalies under RCP 8.5 are even greater and rise beyond 4.5°C in 2080s. | There is Increase and decrease trend in rainfall within the state while the south-western and southern region of the state feels a slight decrease in overall precipitation in 2030s 0.0mm to 0.2mm/day to increase of 0.1mm/day in 2080s. the northern and north-eastern regions suffers a dramatically increase in precipitation up to 0.8mm/day in 2030s to 2080s.In both the RCPs, precipitation increases at highest rate in northern areas as compared to southern areas. |  |

**Rajasthan**


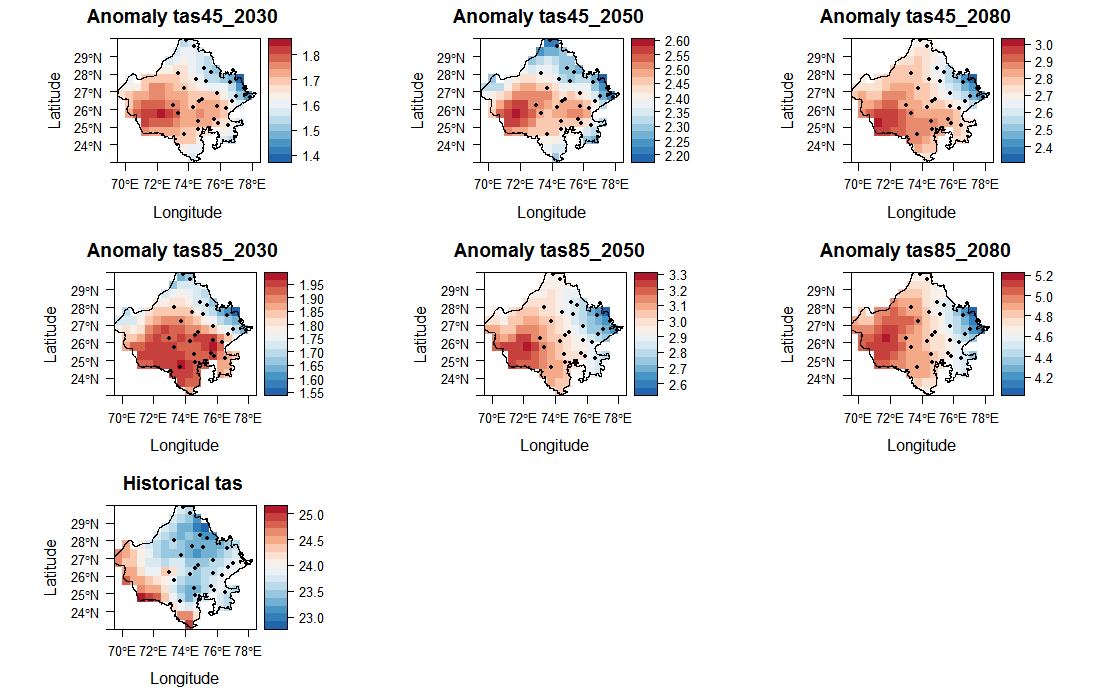


**Map 3.33: Rajasthan– Temperature (Avg.) anomalies (°C) against historical trend**


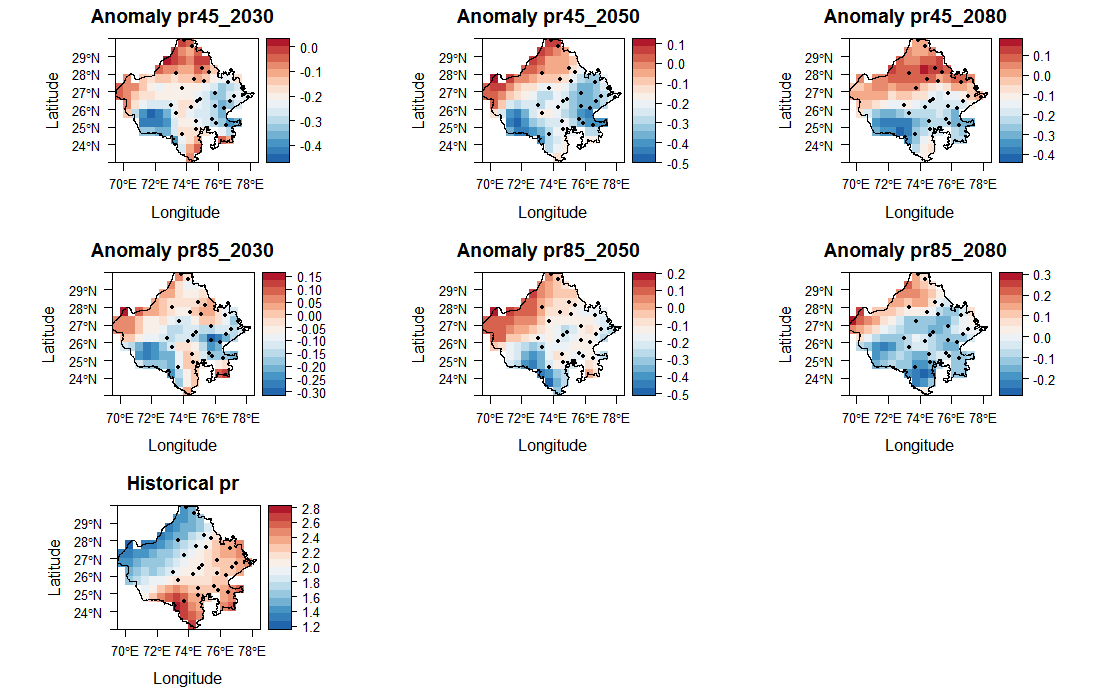


**Map 3.34: Rajasthan – Precipitation anomalies (mm/day) against historical trend**

| **Region/ State** | **Climate variations** | |
| --- | --- | --- |
|  | **Temperature** | **Precipitation** |
| **Rajasthan** | Projections under RCP 4.5 indicate that the temperature is set to rise by 1.4°C- 1.8°C by 2030s to upto 3.0°C by 2080s in the state. While southern-western region of Rajasthan faces highest increase in temperature in both maximum and minimum temperature as compared to Northern and Eastern region of the state. The anomalies under RCP 8.5 are even greater and rise beyond 5.2°C in 2080s. | There is Increase and decrease trend in rainfall within the state, particularly in Northern and Western parts of the state, in long-term scenarios of both RCP4.5 and RCP8.5. The southern part of the state consistently shows a decline in precipitation of 0.4 to 0.1 mm/day in RCP4.5 to 0.2 to 0.05 mm/day in RCP8.5 for 2080s |

**Tamil Nadu**


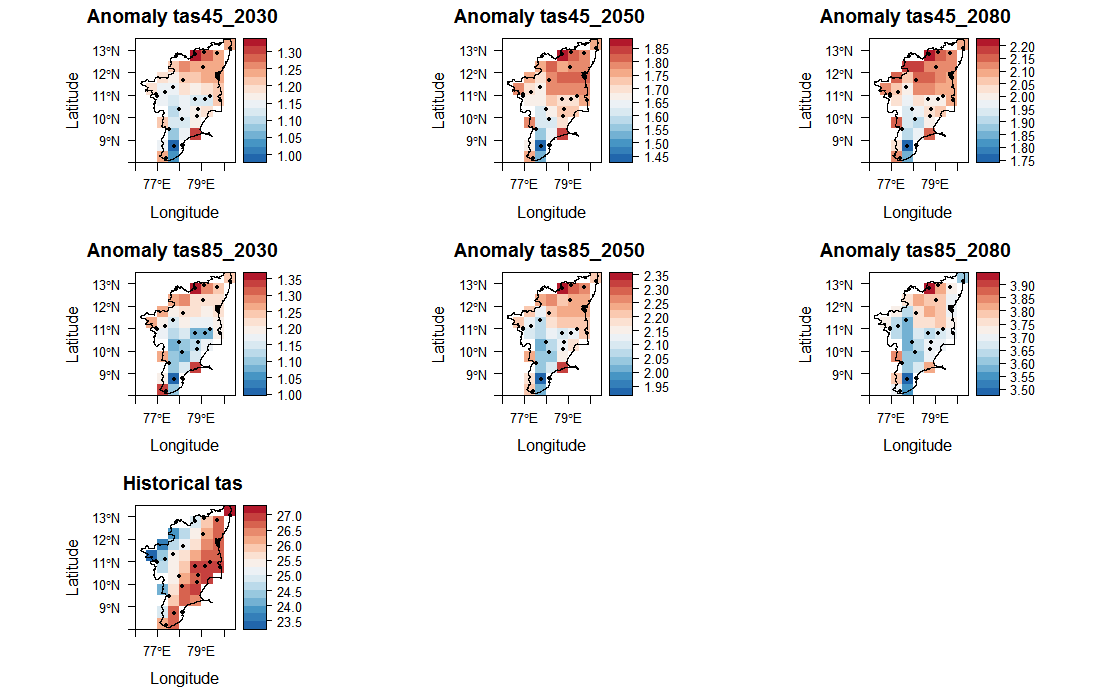


**Map 3.35: Tamil Nadu– Temperature (Avg.) anomalies (°C) against historical trend**


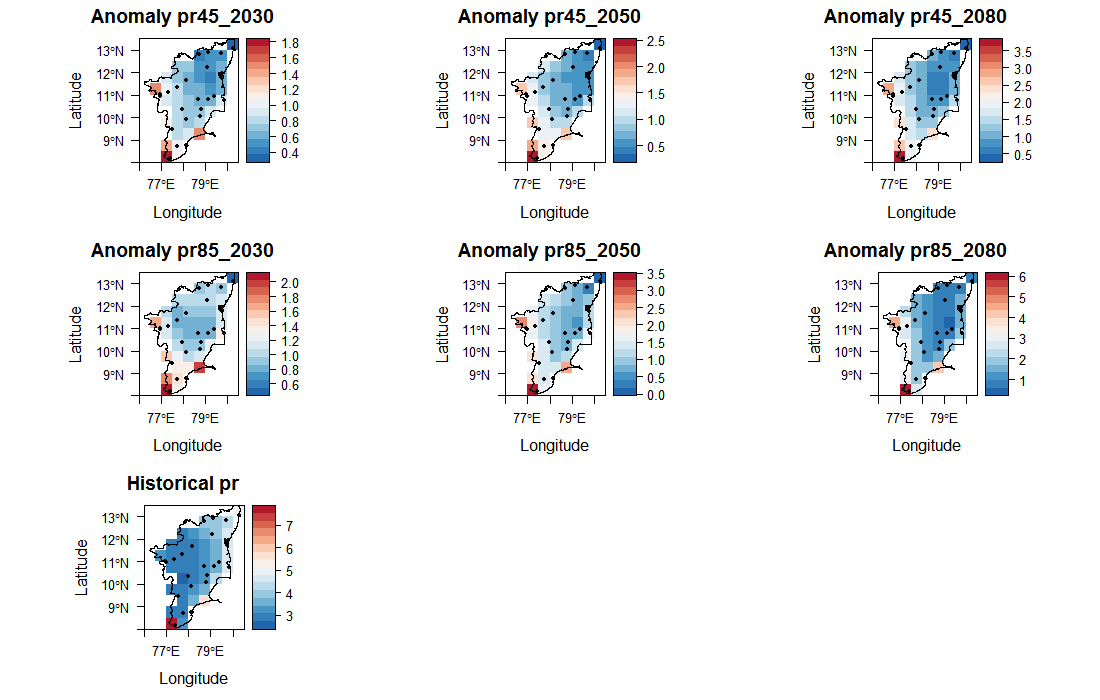


**Map 3.36: Tamil Nadu– Precipitation anomalies (mm/day) against historical trend**

| **Region/ State** | **Climate variations** | |
| --- | --- | --- |
|  | **Temperature** | **Precipitation** |
| **Tamil Nadu & Puducherry** | Average annual temperature increase under both RCP4.5 and RCP8.5 scenario in the region. The rise of temperature ranges from +1.00-1.30°C (2030s), 1.45-1.85°C (2050s) and 1.75-2.20°C (2080s) and for RCP8.5: 1.00-1.35°C (2030s), 1.95-2.35°C (2050s), 3.50-3.90°C (2080s). | As per estimates, under RCP 4.5, precipitation is showing an increase in the region ranging from 0.4-1.8 mm/day (2030s), to 0.5-2.5 mm/day (2050s), to 0.5-3.5 mm/day (2080s). Similarly, RCP 8.5 scenario also shows a significant increase in average precipitation, ranging from 0.6-2.0 mm/day (2030s), to 0.6-3.5 mm/day (2050s), to 1.0-6.0 mm/day (2080s). |

**Uttarakhand**


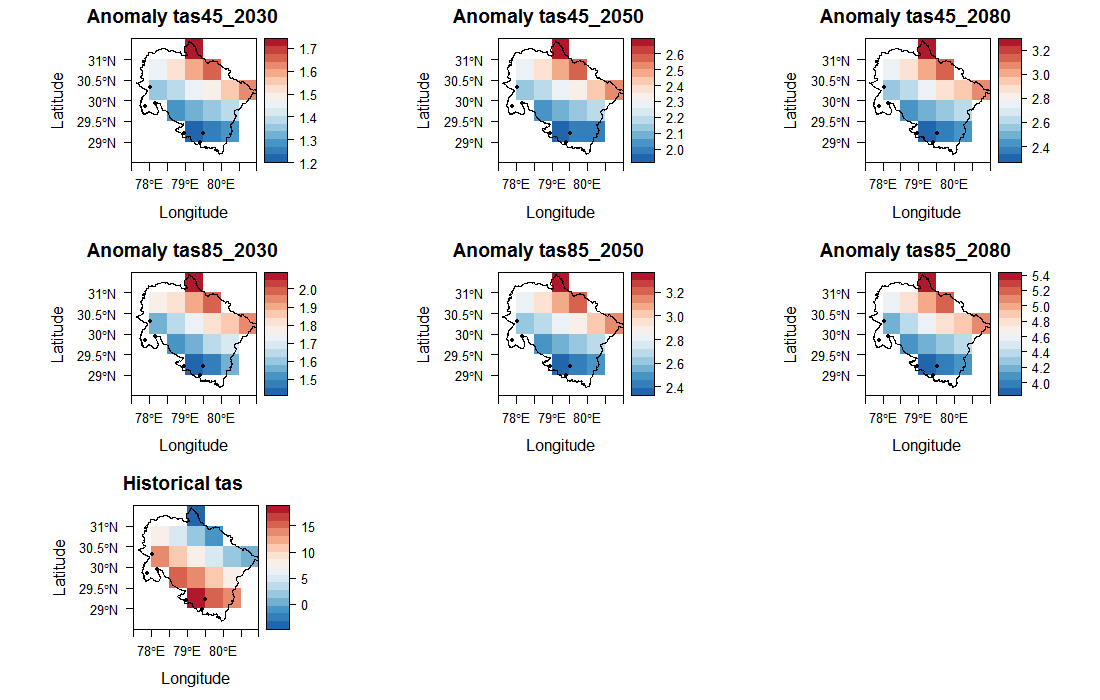


**Map 3.37: Uttarakhand– Temperature (Avg.) anomalies (°C) against historical trend**

**
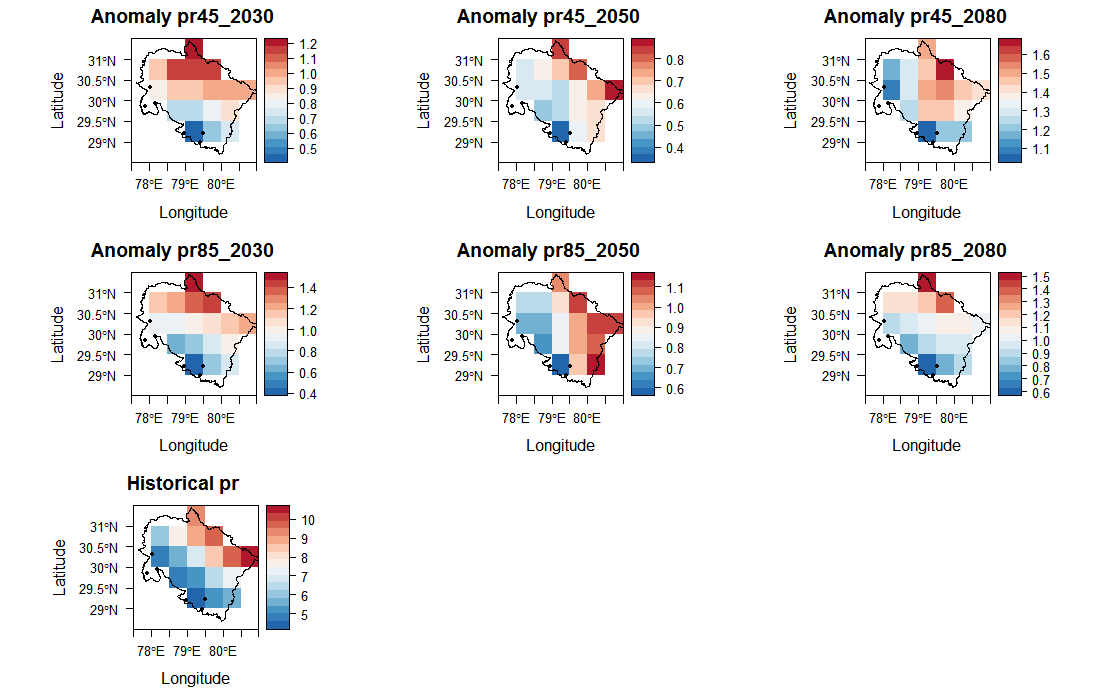
**

**Map 3.38: Uttarakhand– Precipitation anomalies (mm/day) against historical trend**

| **Region/ State** | **Climate variations** | |
| --- | --- | --- |
|  | **Temperature** | **Precipitation** |
| **Uttarakhand** | Projections under RCP 4.5 indicate that the temperature is expected to rise by up to 1.2°C to up to 1.4°C by 2030s to up to 2.6°C in the Terrai and Shivalik region; from 1.4°C to 1.5°C by 2030s to up to 2.8°C by 2080s in the middle Himalayan regions; and from 1.5°C to 1.7C by 2030s to above 2.8°C by 2080s in higher Himalayas. The anomalies under RCP 8.5 are even greater and rise beyond 5°C in higher Himalayas can be expected by 2080s, which is an ecologically sensitive area. | Precipitation is set to increase in the entire state from 0.5 to 1.2mm/day in 2030s to up to 1.1mm/day to 1.6mm/day by 2080s. The increase in precipitation is highest in the higher Himalayan region. |

**Uttar Pradesh**


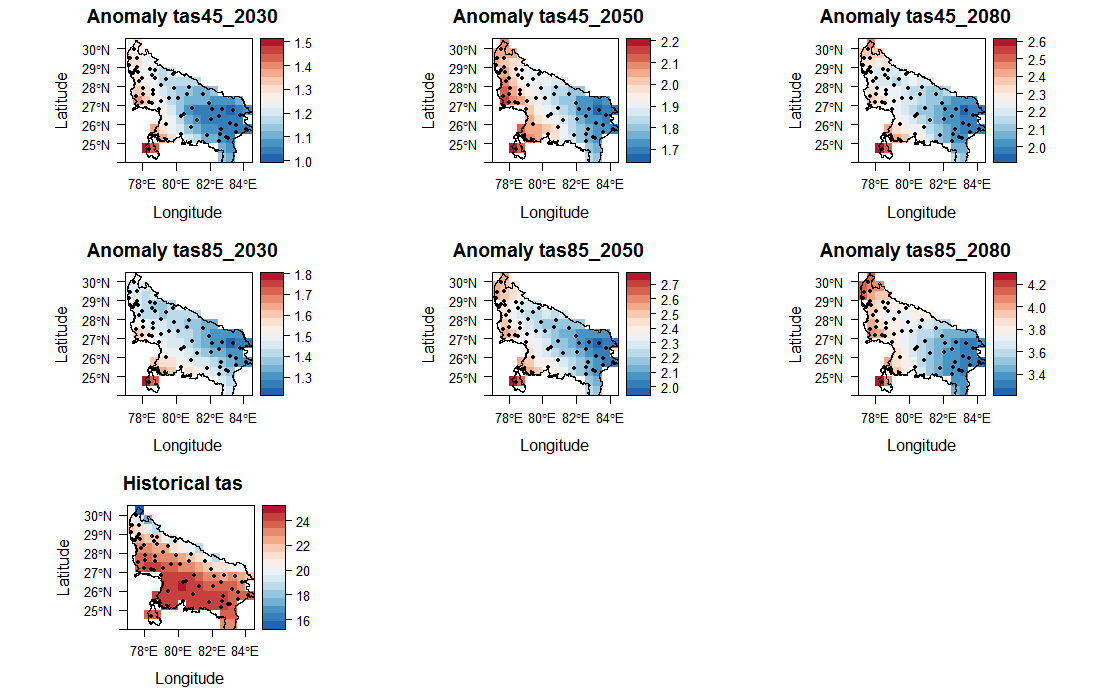


**Map 3.39: Uttar Pradesh– Temperature (Avg.) anomalies (°C) against historical trend**


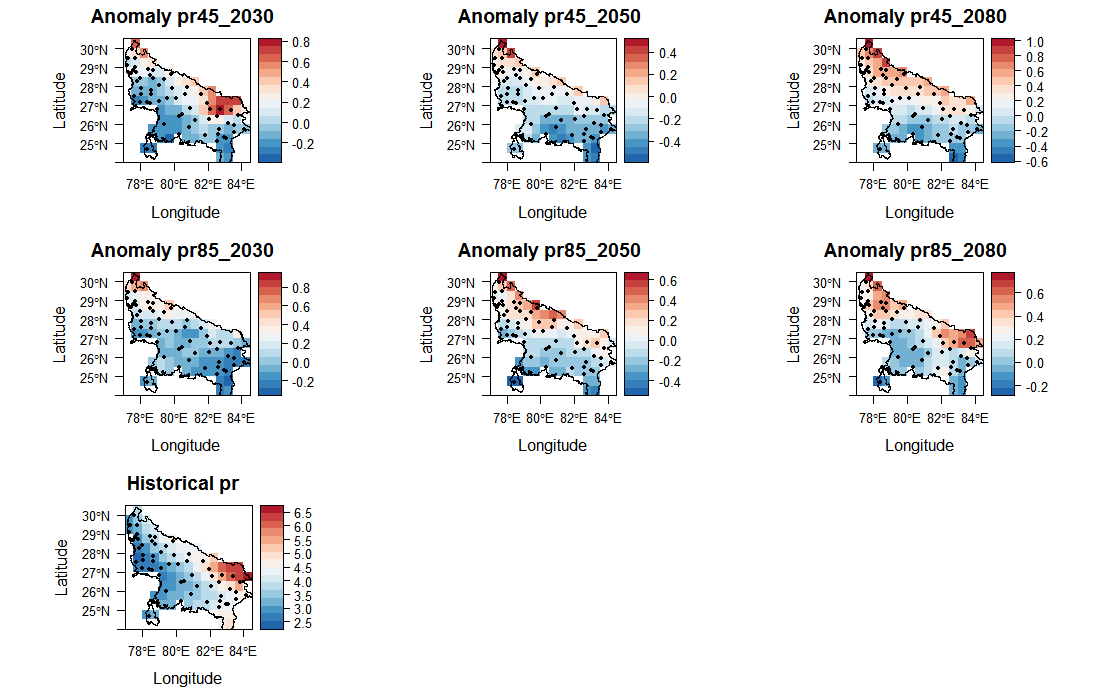


**Map 3.40: Uttar Pradesh– Precipitation anomalies (mm/day) against historical trend**

| **Region/ State** | **Climate variations** | |
| --- | --- | --- |
|  | **Temperature** | **Precipitation** |
| **Uttar Pradesh** | Projections suggest that average annual temperature anomalies are highest (1.20°C -1.50°C in 2030s to 2.20°C-2.60°C in 2080s in RCP4.5 in Bundelkhand region in southern Uttar Pradesh and followed by a rise of upto 2.40°C in 2080s in western Uttar Pradesh. The increase in minimum and maximum temperature is expected to be even more significant in RCP8.5 scenario by 2080s in all parts of the state, ranging from 3.4°C-3.8°C and 3.7°C-4.1°C, respectively. | Precipitation is expected to progressively decline from Terai region along Nepal border towards South & East UP. While Terai region is expected to show an increase of 0.2-0.6 mm/day in RCP4.5 to 0.3-0.7 mm/day in RCP8.5 for 2080s. Amongst all subzones, West UP consistently shows the maximum variations in rainfall pattern from 2030s to 2080s across RCP4.5 and RCP8.5 ranging from -0.2 to 0.7 mm/day. |

**West Bengal**


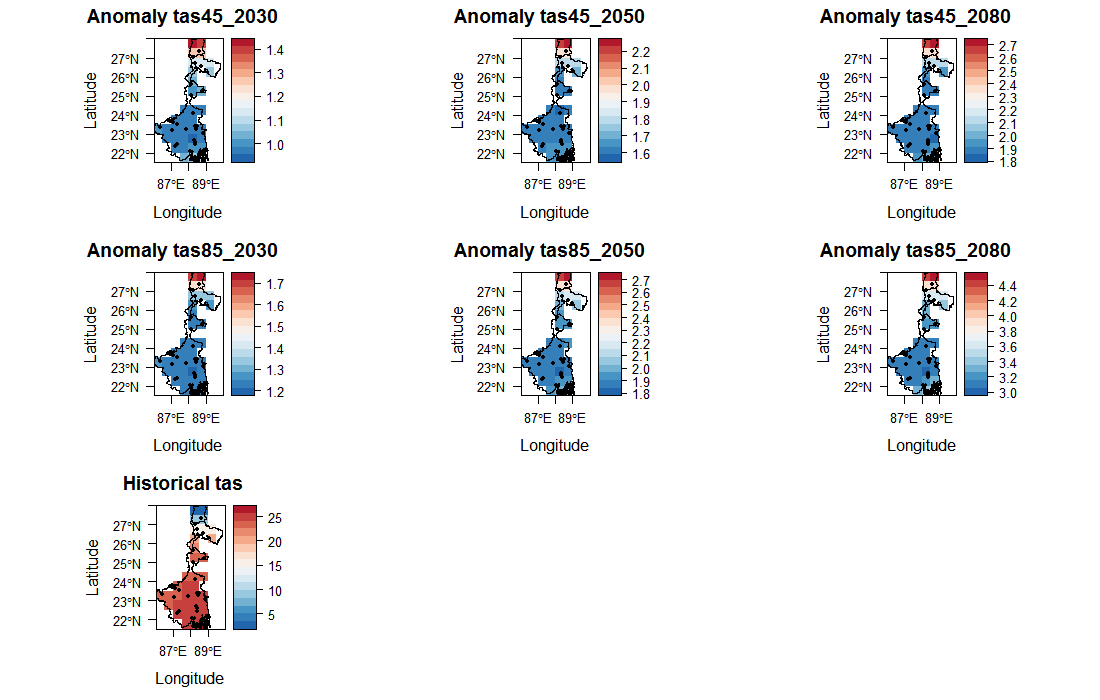


**Map 3.41: West Bengal and Sikkim–Temperature (Avg.) anomalies (°C) against historical trend**

| **Region/ State** | **Climate variations** | |
| --- | --- | --- |
|  | **Temperature** | **Precipitation** |
| **West Bengal** | Estimates for average annual temperature increase under RCP4.5 in the state ranging between 0.95-1.15°C (2030s), 1.50-1.80°C (2050s) and 1.85-2.10°C (2080s) and for RCP8.5: 1.21-1.36°C (2030s), 1.80-2.15°C (2050s), 3.0-3.60°C (2080s). The Northern part of the state, in general, shows marginally greater rise in temperature in both RCP scenarios and for all time positions, than Southern part. | As per estimates, under RCP 4.5, precipitation is showing variable trends in the state ranging from -0.1 to 1.75 mm/day (2030s), to -0.5 to +2.5 mm/day (2050s), to -0.5 to +3.5 mm/day (2080s). RCP 8.5 scenario also shows similar variability in average precipitation, ranging from -0.5 to 1.5 mm/day (2030s), to -0.5 to +3.5 mm/day (2050s), to 0.0 to 1.5mm/day, with extremes of 6.0 mm/day (2080s) in Sunderbans. |
