# Supplementary material for: How to tackle complexity in urban climate resilience? Negotiating climate science, adaptation and multi-level governance in India
Source: PLoS One. 2021 Jul 1;16(7):e0253904. doi: 10.1371/journal.pone.0253904 (PMC8248603; doi:10.1371/journal.pone.0253904)
Supplement: S3 Appendix — (DOCX) [file pone.0253904.s003.docx]

S3 Appendix. Adaptation for Temperature Rise, Heat Waves and Droughts

| **Adaptation Measures** | **Priority^[[1]](#footnote-1)^** | **Implementation time^[[2]](#footnote-2)^** | **Intervention level** |  |
| --- | --- | --- | --- | --- |
| **Water** | | | | |
| Increasing efficiency of operations and reducing water leakages to increase water availability | VH | S | City |  |
| Demand management and reducing wastage of water through consumer education and regulatory provisions | VH | S | City/ Household |  |
| Stopping discharge of wastewater into water bodies and preventing water pollution | VH | S/M | City/ Sub-city |  |
| Rejuvenation of water bodies to recharge ground water and to reduce heat in nearby areas | VH | M | City |  |
| Conjunctive water management | VH | M | City |  |
| Recycling waste water at city level and in all new building complexes; | H | S | City/ building |  |
| Regulate ground water extraction through borewells | H | S | City |  |
| Rainwater harvesting and recharging ground water |  |  |  |  |
| Encourage traditional methods of water storage, e.g., small underground storage ponds, increase the number of local groundwater recharge projects | M | S | City/ Sub-city |  |
| Installation of stationary/ mobile vending stations for potable drinking water | VH | S | City |  |
| **Infrastructure** | | | | |
| Prioritizing solar energy options for public and private buildings | H | S | City |  |
| Energy conservation through measures such as promoting the use of LED lighting and adopting ECBC code for residential apartments and commercial centers | H | S | City |  |
| Promotion of Net Metering for incentivizing solar power generation | H | S | Building/City |  |
| Retrofitting of industrial buildings to make them climate resilient | M | M | City/Sub-city |  |
| Promoting decentralized waste management | H | S | City/ Sub-city/ Neighborhood |  |
| Provisions of scientific landfills | H | M | City |  |
| Integrated municipal solid waste management | H | M | City |  |
| **Building** | | | | |
| Green roofs and Cool roofs (such as the use of reflective paint, China mosaic, hollow clay tile, inverted earthen pots, bamboo shading screen, heat resistance tiles, shading screens, etc.) | H | S | Building |  |
| Implementation of GRIHA rating system; Green Buildings | H | M | City |  |
| Promoting vernacular / traditional construction methods which take into account the local climate conditions | M | M | Building |  |
| **Health** | | | | |
| Developing disease surveillance system for disease outbreaks, improving health and decentralizing health facilities for easy and quick access such as through Primary Health Centres | VH | S | City |  |
| Regular training and drill-preparedness of emergency response teams | H | M | City |  |
| **Urban Planning** | | | | |
| Promotion of urban tree plantation with a view to realize the co-benefits with respect to climate change effects as well as carbon sinks | H | S | City/ Sub-city/ Neighborhood |  |
| Neighbourhood designs to allow better air movement | H | M | Neighbourhood |  |
| Cool pavements | VH | S | City |  |
| Green infrastructure and improving green and blue areas | H | M | City |  |
| Integration of urban and regional plans including climate induced events | M | S | City/ State |  |
| Preventive measures to reduce UHI effect based on scientific mapping of ‘hotspots’, where temperatures are particularly high | M | S | City |  |
| Research studies on urban and/or regional climate change for energy demand, sustainable land use etc. | M/H | S & M | City |  |
| **Additional measures for Heat waves** | | | | |
| Early warning systems with Automatic Weather Stations (AWS) | H | S | City |  |
| Preparation of Heat Action Plan | VH | S | City |  |
| Additional measures such as shading traffic junctions and misting sprinklers | H | S | City |  |
| Providing shelters homes, shelter places with water and first aid | H | M | City |  |

1. Priority: VH=Very High, H=High, M=Medium [↑](#footnote-ref-1)
2. Implementation time: S=Short (Less than 5 yrs), M=Medium (5-10 yrs), L=Long (Over 10 yrs) [↑](#footnote-ref-2)
