# Supplementary material for: How to tackle complexity in urban climate resilience? Negotiating climate science, adaptation and multi-level governance in India
Source: PLoS One. 2021 Jul 1;16(7):e0253904. doi: 10.1371/journal.pone.0253904 (PMC8248603; doi:10.1371/journal.pone.0253904)
Supplement: S4 Appendix — (DOCX) [file pone.0253904.s004.docx]

**S4 Appendix. Adaptation for Heavy Rainfall Events and Flash Floods**

| **Adaptation Measures** | **Priority** | **Implementation time** | **Intervention level** |  |
| --- | --- | --- | --- | --- |
| **Water** | | | | |
| Climate resilient peri-urban agriculture | M | M | City/ State |  |
| Construction of medium and large retention chambers at strategic locations | H | M | City/ Sub-city |  |
| Flood shelters in vulnerable locations and strengthen infrastructure including electrical network that can withstand multiple hazards | VH | S | City |  |
| Ground water recharge and rain water harvesting in buildings, public parks, and paved areas, improving natural depressions to store and regulated excess flow | H | S | City/ Neighborhood |  |
| Insuring lives and assets by the government | M | M | City/ State |  |
| Promoting rain water harvesting arrangements as per building by-laws | H | S | City/ Building |  |
| Raising embankments to prevent spilling/flooding, carrying out channel and drainage improvement works, construction of levees, reservoirs etc. | H | S, M | City/ Sub-city |  |
| Ban construction in high flood risk zones; construct bunds and dykes to prevent flooding; permit flood resistant agriculture on river banks and peri-urban areas | M, H | M | City |  |
| Covering cities with drainage, keeping drains clean and separate from storm water drainage and sewerage system | VH | S | City |  |
| Maintenance and upgradation of drains and other infrastructure to address future flooding events | H | M | City |  |
| **Infrastructure** | | | | |
| Constructing structures such as flood gates, dikes, bulkheads | M | S, M | City/ State |  |
| Standard monitoring of power and telecom lines, equipment, poles and support structures, against extreme rainfall and rising flood levels. | VH | S | City |  |
| Green infrastructure and improving green and blue areas | H | M | City |  |
| Provisions of scientific landfills | H | M | City |  |
| **Buildings** | | | |  |
| Designing building to protect from floods, and related climate events | M | M | Building |  |
| Provide provision for water storage and rain water harvesting | H | M | City |  |
| Shelter homes for homeless in case of extreme climate events | H | S | City |  |
| **Health** | | | | |
| Mobile based apps on disease outbreak with prevention measures | H | S | City |  |
| Disease surveillance system | VH | S | City |  |
| Mobile Urban Health Clinics in strategic locations for optimal outreach | H | S | City |  |
| Regular training and drill-preparedness of emergency response teams | H | M | City |  |
| **Planning** | | | | |
| City Planning based on flood maps - plan for safe zones, green areas, preserving natural drainage and water bodies | H | L | City |  |
| Constructing structures such embankments | M | S, M | City/ State |  |
| Green infrastructure and improving green and blue areas | H | M | City |  |
| City drainage map | H | S | City |  |
| Urban agriculture | M | M |  |  |
| Integration of urban and regional plans including climate induced events (e.g. flooding may originate outside the political and administrative boundaries) | M | S | City/ State |  |
| Research studies on urban and/or regional climate change for energy demand, sustainable land use etc. | M/H | S & M | City |  |
| City Planning based on flood maps - plan for safe zones, green areas, preserving natural drainage and water bodies | H | L | City |  |

Priority: VH=Very High, H=High, M=Medium

Implementation time: S=Short (Less than 5 yrs), M=Medium (5-10 yrs), L=Long (Over 10 yrs)
