# Supplementary material for: How to tackle complexity in urban climate resilience? Negotiating climate science, adaptation and multi-level governance in India
Source: PLoS One. 2021 Jul 1;16(7):e0253904. doi: 10.1371/journal.pone.0253904 (PMC8248603; doi:10.1371/journal.pone.0253904)
Supplement: S5 Appendix — (DOCX) [file pone.0253904.s005.docx]

S5 Appendix. Adaptation for Landslides

| **Adaptation Measures** | **Priority** | **Implementation time** | **Intervention level** |  |
| --- | --- | --- | --- | --- |
|  | | | | |
| Strengthening of slopes for example by afforestation of steep slopes; do not allow construction in landslide prone zones; position key post disaster infrastructure on safer height | H | M | City/ State |  |
| Landslide risk zone maps and landslide inventories to be considered when planning | H | M | City |  |
| Protective measures in landslide prone areas | VH | S | City |  |
| Early Warning System for landslides | H | M | City |  |

Priority: VH=Very High, H=High, M=Medium

Implementation time: S=Short (Less than 5 yrs), M=Medium (5-10 yrs), L=Long (Over 10 yrs)
