# Supplementary material for: How to tackle complexity in urban climate resilience? Negotiating climate science, adaptation and multi-level governance in India
Source: PLoS One. 2021 Jul 1;16(7):e0253904. doi: 10.1371/journal.pone.0253904 (PMC8248603; doi:10.1371/journal.pone.0253904)
Supplement: S6 Appendix — (DOCX) [file pone.0253904.s006.docx]

S6 Appendix. Adaptation for Sea Level Rise

| **Adaptation Measures** | **Priority** | **Implementation time** | **Intervention level** |
| --- | --- | --- | --- |
| Constructing structures such as flood gates, dikes, bulkheads | M | S, M | City/ State |
| Practice beach nourishment that prevents or slows beach erosion and shoreline migration | M | M | City/ State |
| Salt water mapping to protect water supplies | S | M | City/ State |
| Restricting development in coastal erosion areas(e.g. through CRZ norms) | H | S | City |
| Retrofitting houses (to be built on stilts in deltaic and other flood prone areas) | H | S | Building |
| Rain water harvesting to minimize dependence on surface and ground water that are increasingly being intruded by salts from sea water | H | M | City |

Priority: VH=Very High, H=High, M=Medium

Implementation time: S=Short (Less than 5 yrs), M=Medium (5-10 yrs), L=Long (Over 10 yrs)
